# AUF-1 knockdown in mice undermines gut microbial butyrate-driven hypocholesterolemia through AUF-1–Dicer-1–mir-122 hierarchy

**DOI:** 10.3389/fcimb.2022.1011386

**Published:** 2022-12-19

**Authors:** Oishika Das, Jayanta Kundu, Atanu Ghosh, Anupam Gautam, Souradeepa Ghosh, Mainak Chakraborty, Aaheli Masid, Samiran Sona Gauri, Debmalya Mitra, Moumita Dutta, Budhaditya Mukherjee, Surajit Sinha, Moumita Bhaumik

**Affiliations:** ^1^ Department of Immunology, Indian Council of Medical Research-National Institute of Cholera and Enteric Diseases, Kolkata, India; ^2^ School of Applied and Interdisciplinary Sciences, Indian Associations for Cultivation of Science, Kolkata, India; ^3^ Department of Algorithms in Bioinformatics, Institute for Bioinformatics and Medical Informatics, University of Tübingen, Tübingen, Germany; ^4^ International Max Planck Research School “From Molecules to Organisms”, Max Planck Institute for Biology Tübingen, Tübingen, Germany; ^5^ Cluster of Excellence: EXC 2124: Controlling Microbes to Fight Infection, University of Tübingen, Tübingen, Germany; ^6^ School of Medical Science and Technology, Indian Institute of Technology, Kharagpur, India

**Keywords:** AUF-1, butyrate, cholesterol metabolism, Dicer-1, GMO–PMO, microbiome, mir-122.

## Abstract

**Introduction and objective:**

Cholesterol homeostasis is a culmination of cellular synthesis, efflux, and catabolism to important physiological entities where short chain fatty acid, butyrate embodied as a key player. This discourse probes the mechanistic molecular details of butyrate action in maintaining host-cholesterol balance.

**Methods:**

Hepatic mir-122 being the most indispensable regulator of cholesterol metabolic enzymes, we studied upstream players of mir-122 biogenesis in the presence and absence of butyrate in Huh7 cells and mice model. We synthesized unique self-transfecting GMO (guanidinium-morpholino-oligo) linked PMO (Phosphorodiamidate-Morpholino Oligo)-based antisense cell-penetrating reagent to selectively knock down the key player in butyrate mediated cholesterol regulation.

**Results:**

We showed that butyrate treatment caused upregulation of RNA-binding protein, AUF1 resulting in RNase-III nuclease, Dicer1 instability, and significant diminution of mir-122. We proved the importance of AUF1 and sequential downstream players in AUF1-knock-down mice. Injection of GMO-PMO of AUF1 in mouse caused near absence of AUF1 coupled with increased Dicer1 and mir-122, and reduced serum cholesterol regardless of butyrate treatment indicating that butyrate acts through AUF1.

**Conclusion:**

The roster of intracellular players was as follows: AUF1-Dicer1-mir-122 for triggering butyrate driven hypocholesterolemia. To our knowledge this is the first report linking AUF-1 with cholesterol biogenesis.

## Introduction

The gut microbiome is endowed with the special ability to govern host metabolic function, which cannot be accomplished by the host itself. Gut microbiota on fermentation of non-digestible carbohydrates produce short-chain fatty acids (SCFAs) like acetate, propionate, and butyrate at a ratio of 3:1:1 (Chambers et al., 2018). There is colossal evidence that gut-derived SCFAs display overarching effects on health and diseases (Rios-Covian et al., 2016). The benefits of butyrate are particularly well-researched as a therapeutic agent against inflammatory diseases, neurological disorders, and others (Canani et al., 2011). Butyrate is also reported to mediate the host cellular energy metabolic landscape (Liu et al., 2018). Studies in mouse models have shown that a diet supplemented with butyrate prevents high-fat diet (HFD)-induced obesity, by downregulating the expression and activity of PPAR-γ (den Besten et al., 2015), while the intake of dietary polysaccharide pectin that produces butyrate after fermentation in gut inhibits cholesterol uptake and promotes cholesterol efflux from enterocytes in Apo E^-/-^ mice (Chen et al., 2018). There are different types of lipoproteins (HDL, LDL, VLDL, and chylomicron) consisting of different percentages of cholesterol, protein, triglyceride, and phospholipids.

Cholesterol orchestrates a number of important cellular functions like membrane fluidity, steroid hormone synthesis, and bile acid synthesis, while its intermediate metabolic precursor, 7-dehydrocholesterol, serves as a precursor of vitamin D (Zampelas and Magriplis, 2019). Dysregulation of cholesterol metabolism is associated with cardiovascular disease, neurodegenerative diseases, diabetes, and cancer (Merscher-Gomez et al., 2013; Kuzu et al., 2016; Vallejo-Vaz et al., 2017).

Cholesterol homeostasis at the cellular level is governed at multiple layers like biosynthesis, catabolism, and efflux; the latter pathway is accomplished by ATP-binding cassette transporter A1 (ABCA1) (Yancey et al., 2003). These distinctly different, linked paradigms are converged to a singular framework of host cholesterol homeostasis under the influence of butyrate.

At the cellular level, the regulation of cholesterol biogenesis and efflux is governed by microRNAs; the important ones include mir-122 and miR27 (Krutzfeldt et al., 2005; Canani et al., 2011). mir-122, making up 70% of the total hepatic miRNA (Lagos-Quintana et al., 2002), contributes to the maintenance of the adult liver phenotype (Girard et al., 2008). Mice treated with antagomir-122 were shown to modulate cholesterol metabolic genes (Krutzfeldt et al., 2005). Like most miRNAs, mir-122 is generated from the primary precursor, pri-mir-122, by nuclear RNaseIII Drosha; the resultant pre-mir-122 undergoes processing with the help of Dicer-1, another RNase III nuclease that processes the pre-miRNA into a 22-bp double-stranded RNA (O’Brien et al., 2018). Dicer-1-mediated mir-122 maturation is a critical step in mir-122 biogenesis, which occurs in the cytoplasm.

In mammals, the Dicer-1 mRNA is destabilized by the presence of AU-rich elements (AREs) in their 3’-untranslated regions (Abdelmohsen et al., 2012). The protein ARE/poly(U)-binding/degradation factor-1 (AUF-1) binds to many ARE-mRNAs and assembles other factors necessary to recruit the mRNA degradation machinery (Gratacos and Brewer, 2010). AUF-1 RNA binding proteins are a family with four splice variants: AUF-1^p45^, AUF-1^p42^, AUF-1^p40^, and AUF-1^p37^ (Abdelmohsen et al., 2012). The AUF-1 interacts with the 3′-UTR of Dicer-1 mRNA and causes mRNA destabilization, reducing its half-life (Abdelmohsen et al., 2012). AUF-1 KO mice develop chronic dermatitis (Sadri and Schneider, 2009) and are prone to septicemic shock (Lu et al., 2006). Importantly, AUF-1 is found to decay mRNA of sphingosine kinase1 (Sobue et al., 2008), which produces Spingosine-1-phospahte (S1P), a biomarker of sepsis (Hasan and Karst, 1989) and other inflammatory diseases (Suh and Saba, 2015). The absence of AUF-1 thus increases S1P, which causes rapid expansion of inflammatory signals (Graler, 2012).

This study determines that gut-derived butyrate flux affects metabolic phenotype through miRNAs, Dicer-1, AUF-1, and cholesterol metabolic enzymes and controls cholesterol homeostasis. We explored domains like expression of cholesterol synthesis, catabolic genes, and efflux protein in the presence and absence of butyrate. We showed that butyrate modulates selective isoforms of RNA binding protein AUF-1, leading to downregulation of Dicer-1 and mir-122, resulting in decrease in cellular cholesterol. To provide further leverage in our understanding, we had synthesized a unique self-transfecting GMO (guanidinium morpholino oligos)-linked PMO (phosphorodiamidate morpholino oligos)-based antisense reagent, to knock down AUF-1 in mice. Using AUF-1 knockdown mice, we provided crucial evidence that butyrate indeed exploits AUF-1 to accomplish its cellular effect. The above discourse led to a new element of our understanding on the role of AUF-1 in cholesterol metabolism. Narratives from our experimental studies when considered in tandem culminated in the following trajectory of butyrate action “butyrate–AUF-1–Dicer-1–mir-122–cholesterol metabolic enzymes–cholesterol”, which is tentatively posited as “axis”.

## Results

### Butyrate effectively reduces cellular cholesterol at lower concentration than propionate and acetate in murine primary hepatocytes but requires higher concentration to reduce cholesterol and HMGCR expression in Huh7 cells

To witness which one of the SCFAs may interfere in cholesterol biogenesis with fidelity, we undertook an initial approach in the primary hepatocyte culture, although most of our obvious thrust is in the mouse model. To assess the effects of SCFAs on cellular cholesterol biogenesis, hepatocytes were treated with increasing concentration (0–0.045 mM) of either sodium butyrate (butyrate) or sodium propionate (propionate) or sodium acetate (acetate) for 24 h, following which cellular cholesterol was measured. The concentration of SCFAs used in the study was based on the concentration reaching the liver *via* portal vein (Cummings et al., 1987). It was observed that unlike propionate and acetate (**Figure S1**), 5, 15, and 45 µM butyrate treatment caused 27%, 36%, and 40% decrease in cellular cholesterol, respectively (**Figure S1A** inset). As primary hepatocytes die in a couple of days in culture and highly prone to dedifferentiation (Hu and Li, 2015), we extended our *in vitro* study in Huh7 cells, which is considered essentially as a model for studying hepatic processes. Interestingly, butyrate treatment at lower doses did not show significant reduction in cholesterol in Huh7 cells, but higher doses (5–20 mM) of butyrate treatment caused a significant decrease in cellular cholesterol. The higher concentration of SCFAs used to treat Huh7 cells was based on an earlier report (Zhao et al., 2021). To ensure the viability of the cells at higher doses of butyrate, we performed an MTT assay where we show that more than 85% of Huh7 cells were viable at 20 mM butyrate (**Figure S2** inset). Since butyrate but not the others reduced cellular cholesterol, we studied the ability of butyrate on the rate-limiting enzyme of the cholesterol biogenesis pathway and opted to study the status of HMGCR (3-hydroxy-3-methylglutaryl-CoA reductase) by Western blot in butyrate-treated and untreated Huh7 cells. It was observed that there was a significant decrease in HMGCR in butyrate-treated cells as compared to untreated cells as a function of butyrate concentration (**Figure S1B**). The results were presented by densitometric analysis (**Figure S1C**). The percentage decrease in HMGCR in Huh7 cells upon the addition of 10 and 20 mM butyrate was 37% and 74%, respectively. Based on the cell line results, we asked the same question in the animal model.

### Butyrate corrects hypercholesterolemia in mice receiving HFD (HFD mice) and reverses elevated hepatic enzymes

The mice were fed with HFD for 30 days and then divided into two groups. One group of mice continued to receive HFD (HFD mice) and another group received butyrate with HFD for another 15 days (HFD butyrate mice). A third group of mice kept as control received regular chow diet (chow mice). All three groups were sacrificed on day 45 to study a variety of parameters. To start with, body weight and food intake of all three groups of mice were recorded. It was observed that in HFD mice, the body weight continued to increase at a higher rate as compared to chow mice up to the 30-day treatment protocol. With the commencement of butyrate treatment on day 31 in HFD mice for another 15 days (HFD butyrate mice), there was a significant decrease in body weight as compared to HFD mice. In other words, on day 45, there was hardly any difference noticed in body weight between normal and HFD butyrate mice (**Figure S2A**, inset). It was observed that all three groups showed a similar magnitude of food intake (**Figure S2B**). Then, we went on to study the status of cholesterol and lipoproteins in all the three groups.

**Figure 1:**
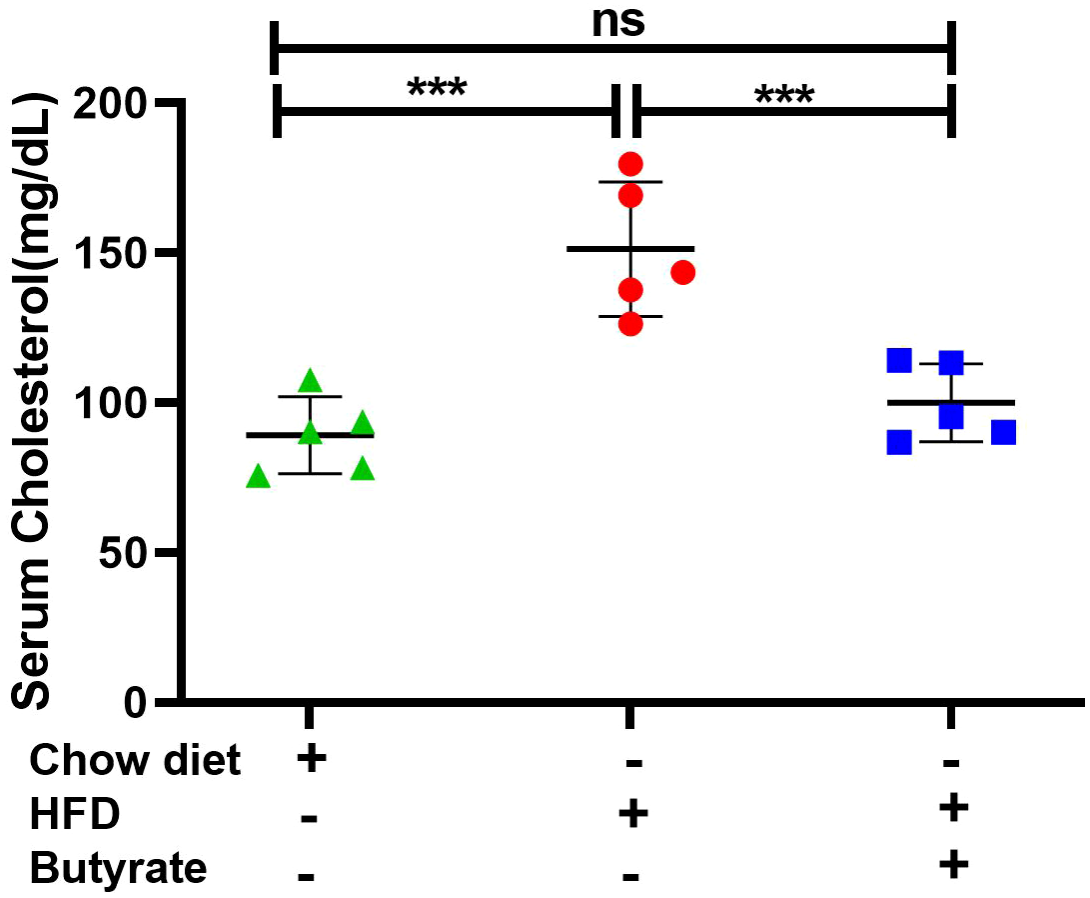
Effect of butyrate treatment on serum cholesterol level of HFD (high-fat diet) fed mice (HFD **mice).** Female mice (10 in number) were fed with HFD for 30 days and then divided randomly into two groups equally. One group continued receiving HFD (HFD mice) and another group received 5% sodium butyrate (butyrate) supplemented with HFD (w/w) for another 15 days (HFD butyrate mice). Another independent group of mice (five in number) were fed with normal chow diet for 45 days, which served as a control (chow mice). On day 45, serum cholesterol levels in chow mice, HFD mice, and HFD butyrate mice were determined and expressed in mg/dl. *N* = 5/group; the experiment was repeated thrice. The data are presented as mean ±SE. ns represents not significant, *** represents *p* < 0.001.

It was observed that compared to chow mice, HFD mice showed a 60% increase in serum cholesterol, which returned to normal in HFD butyrate mice (**Figure 1**). Two liver-specific enzymes, alanine transaminase (ALT) and aspartate aminotransferase (AST), were studied as markers of HFD-induced hepatic stress. It was observed that both ALT and AST were significantly increased in HFD mice, which returned to normal in HFD butyrate mice (**Figure S2C**).

### Butyrate influences cholesterol metabolic genes

The microarray datasets comparing butyrate-treated colonic epithelial cells (MCE301) vs. untreated control (GSE4410) and HeLa cells treated with butyrate vs. corresponding untreated control (GSE45220) are available in public domain. The results were presented in volcano plots and gene expression showing that a statistically significant fold change revealed an array of information on cholesterol metabolic genes (**Figure S3A**). Differential expression analysis indicated that butyrate treatment resulted in an overall repression of cholesterol metabolizing genes, of which some are directly and some are indirectly involved. The common genes between two datasets that were considered for further studies included HMG CoA reductase (*hmgcr*), acetyl CoA acetyltransferase-2 (*acat2*), 7-dehydrocholesterol reductase (*dhcr7*), HMG CoA synthase 1 (*hmgcs1*), and 24-dehydrocholesterol reductase (*dhcr24*). A significant downregulation in *hmgcr*, *acat2*, *dhcr7*, *hmgcs1*, and *dhcr24* expression with butyrate treatment was observed (**Figure S3B**). By enriching our initial data derived from two independent cell lines, our study was expanded to chow mice, HFD mice, and HFD butyrate mice to study the expression of hepatic cholesterol metabolic genes by qPCR.

### Butyrate treatment to HFD mice alters cholesterol metabolic gene expression and decreases lipid droplet formation in the liver

**Figure 2:**
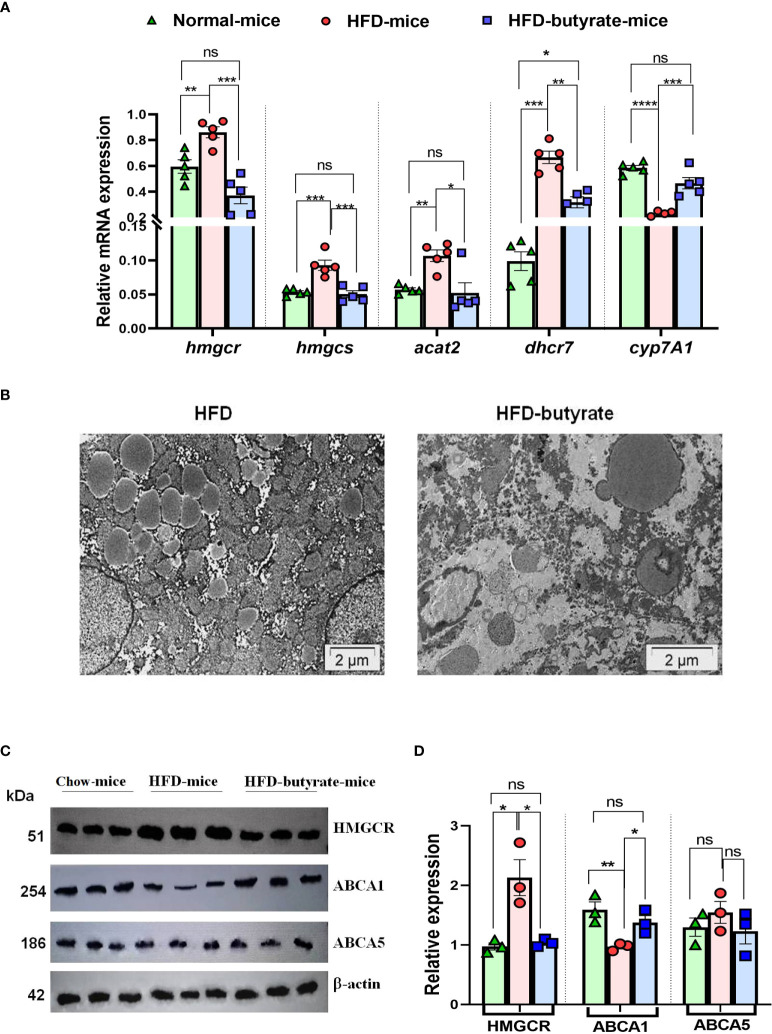
Analysis of hepatic expression of cholesterol metabolic genes by qPCR, lipid droplet status in the liver, and HMGCR, ABCA1, and ABCA5 expression by Western blot in chow mice, HFD mice, and HFD butyrate mice. Hepatic expression of cholesterol metabolic pathway: *hmgcr*, *hmgcs*, *dhcr7*, *acat-2*, and *cyp7A1* were studied in all the groups by qPCR **(A)**. TEM images of the liver sections showing lipid droplets. The sections were visualized under a FEI Tecnai 12 Biotwin transmission electron microscope (FEI, Hillsboro, OR, USA) at an accelerating voltage of 100 kV. Scale bar in the image is 2 µm **(B)**. Analysis of hepatic expression of HMGCR, ABCA1, and ABCA5 by Western blot **(C)**. Densitometry was done using ImageJ showing relative expression with respect to β-actin control **(D)**. *N* = 5/group; data are presented as mean ±SE. The experiment was repeated thrice. **** represents p<0.0001, *** represents *p* < 0.001, ** represents *p* < 0.01, * represents *p* < 0.05, ns represents not significant.

Based on the results from cell lines as described in the volcano plot, we studied hepatic expression of *hmgcr, hmgcs*, *acat2*, and *dhcr7* by qPCR. There was about a twofold increase in hepatic *hmgcr* expression in HFD mice compared to chow mice, which was returned to normal level in HFD butyrate mice (**Figure 2A**). It was observed that in HFD mice, there was a significant increase in hepatic expression of *hmgcs*, *acat2*, and *dhcr7* as compared to chow mice, while the expression of only *hmgcs* and *acat2* returned to normal in HFD butyrate mice. The expression of *dhcr7* in HFD butyrate mice decreased significantly compared to HFD mice, but its expression remained significantly higher compared to chow mice. In addition, we opted to study the expression of the cholesterol catabolic enzyme, which did not surface in the volcano plot: 7A1 of the cytochrome P450 family (*cyp7a1)*. *cyp7a1* expression was significantly reduced in HFD mice compared to chow mice and returned to the normal level in HFD butyrate mice (**Figure 2A**).

The liver sections from the mice were inspected by TEM and the representative images of sections were analyzed, highlighting the preponderance of lipid droplets. It was observed that there was an abundance of lipid droplets of an average diameter of ~1.2 µm in HFD mice whereas in HFD butyrate mice, the droplets were significantly reduced not only in number but also by size with an average diameter of ~0.7 µm (**Figure 2B**).

Since HMGCR is a key regulatory enzyme of cholesterol biogenesis, the results observed in the qPCR study were further validated by Western blot. It was observed that there was twofold increase in the expression of HMGCR in HFD mice compared to chow mice, while the expression returned to normal in HFD butyrate mice (**Figures 2C, D**).

The hepatic expression of proteins ABCA1 (ATP binding cassette subfamily A member 1) and ABCA5 (ATP binding cassette subfamily A member 5) was studied by Western blot (**Figure 2C**).

Densitometry analysis revealed that there was a significant decrease in ABCA1 expression in HFD mice compared to chow mice, and it returned to normal status in HFD butyrate mice (**Figure 2D**). Notably, the expression of ABCA5 remained unchanged in all the three groups (**Figures 2C, D**). For further functional analysis of efflux proteins, we used the Huh7 cell line.

### Butyrate upregulates ABCA1 but not ABCA5 expression and increases cholesterol efflux in Huh7 cells

Butyrate treatment of Huh7 cells showed progressive increase in ABCA1 expression as a function of butyrate concentration as evident from the Western blot (**Figure S4A**) and its corresponding densitometry. On the other hand, expression of ABCA5 remained unaltered regardless of butyrate treatment (**Figure S4B**).

To study efflux, we used 22-NBD-cholesterol-loaded Huh7 cells treated with or without butyrate. The 22-NBD-cholesterol-loaded cells were kept for 18 h in medium before butyrate treatment to allow aqueous phase diffusion of cholesterol. It was observed that in the absence of HDL, regardless of butyrate treatment, there was negligible efflux of cholesterol. Interestingly, there was a significant increase in cholesterol efflux from 22-NBD-cholesterol-loaded Huh7 cells as a function of HDL concentration, which was significantly enhanced while cells were pretreated with butyrate (**Figure S4C**).

### Butyrate downregulates mir-122 biogenesis in Huh7 cells

**Figure 3:**
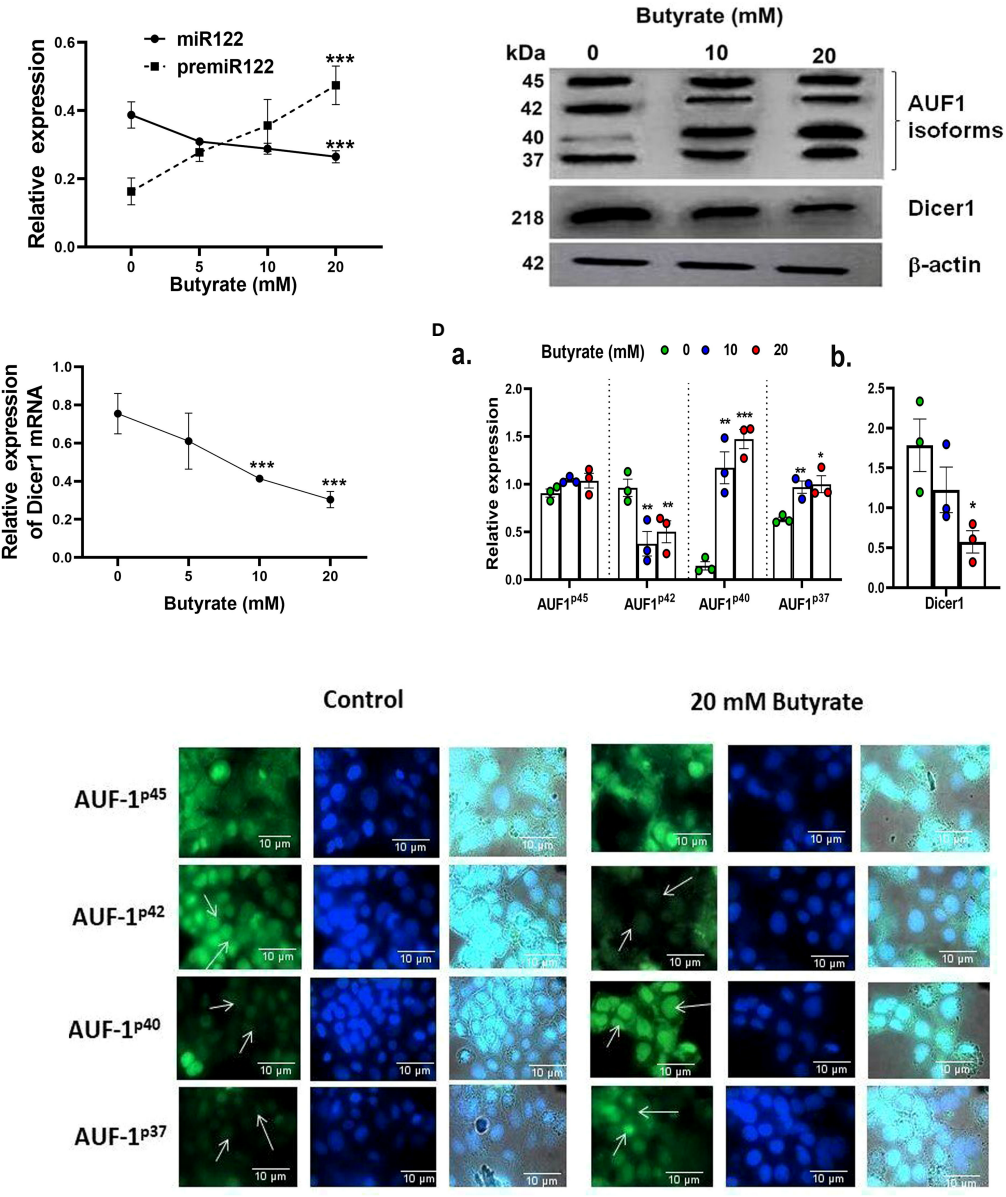
Effect of butyrate on miR122 maturation, Dicer1 expression, AUF1 isoform expression by Western blot, and EGFP reporter assays in Huh7 cells. Expression of pre-miR122 and miR122 **(A)** and Dicer1 **(B)** as determined by qPCR and Western blot of AUF1 isoforms (upper panel) and Dicer1 protein (middle panel) as a function of butyrate concentration **(C)** and corresponding densitometry of AUF1 isoforms (AUF1^p45^/AUF1^p42^/AUF1^p40^/AUF1^p37^) **(D, a)** and Dicer1 **(D, b)** with respect to β-actin control using ImageJ. Transfection of Huh7 cells with individual AUF1 isoforms of EGFP reporter construct (pEGFP-AUF1^p45^ or pEGFP-AUF1^p42^ or pEGFP-AUF1^p40^ or pEGFP-AUF1^p37^) in the absence and presence of 20 mM butyrate for 24 h **(E)**. The resulting fluorescence images are taken under 60× in Apotome Zeiss microscope with a CCD camera controlled with ZEN software (Carl Zeiss, Gottingen, Germany). *N* = 3. The data are presented as mean ± SE. *** represents *p* < 0.001, ** represents p<0.01, * represents *p* < 0.05.

We then monitored the pre-mir-122, mir-122, and Dicer-1 status in Huh7 cells by qPCR. It was observed that there was a decrease in mir-122 (**Figure 3A**) and Dicer-1 (**Figure 3B**) with a concurrent increase in pre-mir-122 (**Figure 3A**) expression as a function of butyrate concentration. We also observed that propionate and acetate at 20 mM did not show any difference in premir-122, mir-122, and Dicer-1 with respect to control (**Figure S4D**).

### Butyrate upregulates AUF-1 and downregulates Dicer-1 and Sphingosine kinase 1 (a classical target of AUF-1) in Huh7 cells

We studied the expression of the posttranscriptional regulator of Dicer-1, AUF-1, in butyrate-treated Huh7 cells using Western blot (**Figure 3C**). The basal expression of AUF-1 isoforms in Huh7 cells was initially p45>p42>p37>p40 which altered to p40>p45>p37>p42 due to butyrate treatment. From the densitometry analyses, it was clear that the expression of AUF-1^p45^remained unaltered whereas that of AUF-1^p42^ significantly decreased due to butyrate treatment with respect to the untreated control. On the other hand, there was an upregulation of the other two isoforms due to butyrate treatment. The fold increase for AUF-1^p40^ after the addition of 10 and 20 mM of butyrate was 7.36 and 8.7, respectively, with respect to the control. On the other hand, there was a marginal increase in AUF-1^p37^ expression after 10 mM butyrate addition and a low but significant increase after 20 mM of butyrate supplementation (**Figure 3D**). Furthermore, there was a progressive decrease in Dicer-1 as a function of butyrate concentration, which was fourfold in the case of 20 mM butyrate-treated Huh7 cells (**Figure 3D**).

We subsequently validated the results by transfection of Huh7 cells with EGFP constructs of each of the AUF-1 isoforms in the presence and absence of 20 mM butyrate (**Figure 3E**). We show that butyrate treatment of Huh7-transfected cells resulted in a decrease in EGFP-AUF-1^p42^ fluorescence and an increase in EGFP-AUF-1^p40^ and EGFP-AUF-1^p37^ fluorescence within transfected cells. Conversely, EGFP-AUF-1^p45^ fluorescence remained essentially unaltered with or without butyrate treatment. As the fluorescent-fused cDNAs were expressed from plasmid promoters, differences in fluorescence would not be due to differences in transcription or splicing but rather due to differences in the stability of mRNA or protein. It is also worth mentioning that unlike butyrate, propionate and acetate did not show any significant increase in AUF-1^p40^ expression as studied by qPCR (**Figure S4D**).

Since AUF-1 is mRNA decay-promoting protein, for which there may be other important targets other than Dicer-1, we also studied the expression of sphingosine kinase (Sphk1). The expression of sphingosine kinase 1 (Sphk1) mRNA, which is another target of AUF-1 (Sobue et al., 2008), was also decreased upon butyrate treatment. We also observed a significant decrease in Sphk1 expression by butyrate treatment to Huh7 cells (**Figure S5**). The reciprocal relation of AUF-1 and Sphk1 expression upon butyrate treatment offered credence to the notion that butyrate acts by targeting AUF-1.

### AUF-1 silencing increased Dicer-1 and mir-122 expression in Huh7 cells

**Figure 4:**
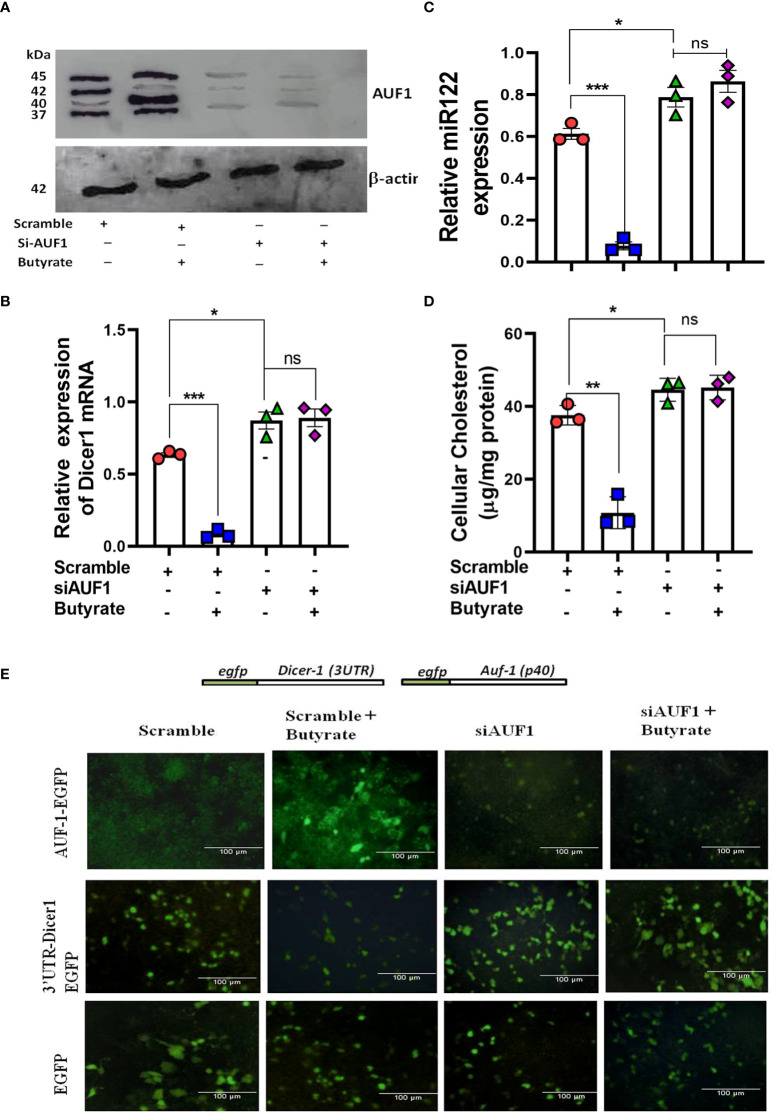
Silencing of AUF1 by siRNA-enhanced miR122, Dicer1 expression, and cellular cholesterol in Huh7 cells regardless of butyrate treatment. Western blot of AUF1 isoforms after co-transfecting with either siAUF1 or scramble-RNA (scramble) where β-actin is used as control **(A)**. The corresponding expression of Dicer1 **(B)** and miR122 **(C)** as analyzed by qPCR and cellular cholesterol in cell lysate **(D)**. Analysis of EGFP expression upon transfection with reporter construct of EGFP-AUF1^p40^, EGFP-3ÚTR-Dicer1, and EGFP (from top to bottom) in a co-transfection experiment under the following conditions: **(i)** scramble, **(ii)** scramble plus 20 mM butyrate, **(iii)** siAUF1 alone, and (iv) siAUF1 plus 20 mM butyrate (from left to right) **(E)**. Resulting fluorescence images of co-transfected cells were captured in 20× magnification in a Carl Zeiss microscope equipped with a CCD camera controlled with ZEN software (Carl Zeiss, Gottingen, Germany). *N* = 3; the data are presented as mean ± SE. *** represents *p* < 0.001, ** represents *p* < 0.01, * represents *p* < 0.05, and ns represents not significant.

The importance of AUF-1 on the expression of Dicer-1 and mir-122 was studied by silencing AUF-1 in Huh7 cells to which the following treatments were added: scramble siRNA (scramble), scramble plus butyrate, siAUF-1 alone, or siAUF-1 plus butyrate. The expression profile of AUF-1 was analyzed by Western blot (**Figure 4A**). Interestingly, silencing AUF-1 with siAUF-1 led to reduction in all the isoforms of AUF-1 regardless of the presence or absence of butyrate (**Figure 4A**). Other cellular consequences due to AUF-1 silencing like the expression of Dicer-1, mir-122, and cellular cholesterol status were also studied. It was observed that silencing with si-AUF-1 resulted in a significant increase in Dicer-1 (**Figure 4B**), mir-122 (**Figure 4C**), and cellular cholesterol (**Figure 4D**) regardless of butyrate treatment, but this was not seen when the cells were transfected with scramble siRNA. Since the upregulation of AUF-1^p40^ was quite prominent of all the AUF-1 isoforms due to butyrate treatment, the rest of the studies were carried out with the AUF-1^p40^ isoform.

To show that AUF-1 repressed Dicer-1 by binding to the 3’-UTR of its mRNA, a co-transfection experiment was performed. Huh7 cells were transfected with either pEGFP-AUF-1^40^, p-EGFP-3ÚTRDicer-1, or pEGFP, and the resulting EGFP fluorescence was analyzed by fluorescence microscopy. Following the second round of transfection with either scramble or siAUF-1, the cells were treated with or without butyrate generating four experimental sets from left to right as follows: (i) scramble, (ii) scramble plus butyrate, (iii) siAUF-1, and (iv) siAUF-1 plus butyrate. From top to bottom, transfection was done with pEGFP-AUF-1^40^, p-EGFP-3ÚTRDicer-1, or pEGFP, respectively.

In the first panel pEGFP-AUF-1^p40^, fluorescence was observed when co-transfected with scramble, which increased after butyrate treatment. On the other hand, the fluorescence of EGFP-AUF-1^p40^ was diminished with siAUF-1, and the effect remained the same even after butyrate treatment. In the second panel for the p-EGFP-3ÚTR Dicer-1 probe, there was a basal level of fluorescence due to scramble siRNA, which diminished after butyrate treatment. The fluorescence of EGFP-3ÚTRDicer-1 was increased with siAUF-1, but remained essentially unchanged after butyrate treatment. The third panel showed that the EGFP expression remained the same during the course of the above treatments (**Figure 4E**). So far, the relation among butyrate–AUF-1–Dicer-1–mir-122 was studied in Huh7 cells. We further translated these findings in a mouse model.

### Hepatic expression of mir-122, Dicer-1, and AUF-1^p40^ in chow mice, HFD mice, and HFD butyrate mice

**Figure 5:**
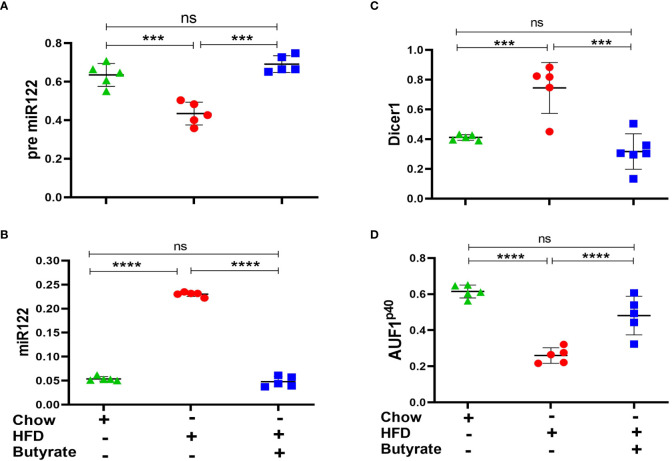
Hepatic miR122, Dicer1, and AUF1^p40^ in chow mice, HFD mice, and HFD butyrate mice. Hepatic expression of pre-miR122 **(A)**, miR122 **(B)**, Dicer1 **(C)**, and AUF1^p40^ **(D)**, in chow mice, HFD mice, and HFD butyrate mice by qPCR. *N* = 5/group; data are presented as mean ±SE. The experiment was repeated thrice. **** represents *p* < 0.0001, *** represents *p* < 0.001, and ns represents not significant.

It was observed that there was a significant decrease in pre-mir-122 with an increase in mir-122 in HFD mice as compared to chow mice. This expression returned to normal in HFD butyrate mice (**Figures 5A, B**). It was also observed that there was an increase in Dicer-1 (**Figure 5C**) with a concomitant decrease in AUF-1^p40^ (**Figure 5D**) in HFD mice and was restored to normal levels in HFD butyrate mice.

### Butyrate downregulates other microRNAs

Since there are reports that mir27 plays an important role in lipid metabolism, we also studied hepatic expression of miR27a and miR27b in chow mice, HFD mice, and HFD butyrate mice. We showed that there was a sixfold increase in hepatic miR27a expression in HFD mice compared to chow mice, which returned to normal levels in HFD butyrate mice. Interestingly, miR27b remained unaltered in all the three groups (**Figure S6**).

### Oral butyrate or probiotic treatment increases fecal butyrate and reduces serum cholesterol in antibiotic-treated mice following the AUF-1–Dicer-1–mir-122 pathway; probiotic treatment restores gut butyrate-producing bacteria

**Figure 6:**
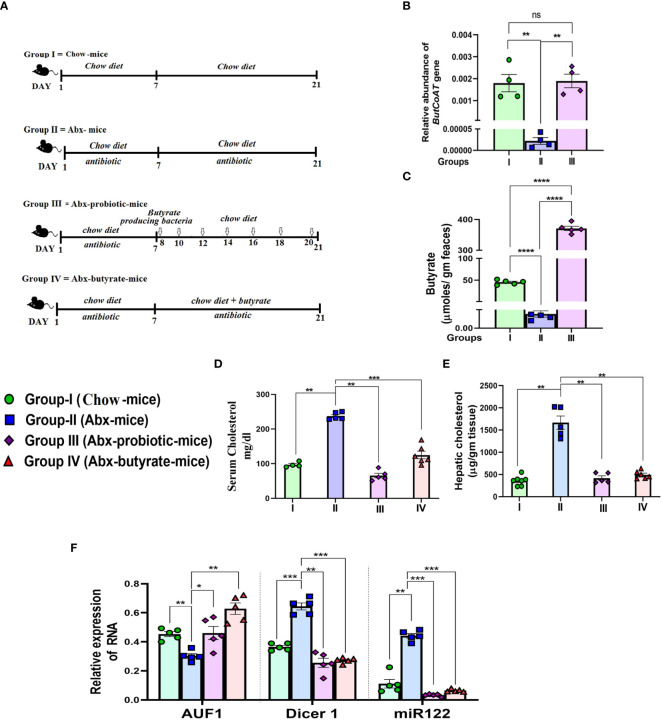
Effect of antibiotic treatment and subsequent probiotic treatment on fecal butyrate, serum and cholesterol, AUF1p40, Dicer1, and miR122 expression. Female mice were used for the study. Experimental design of the study: Group I = untreated chow-fed mice (untreated mice). Group II = mice receiving cocktail antibiotic and chow diet for 21 days (Abx mice). Group III = mice receiving cocktail antibiotic for the first 7 days followed by bowel cleansing by PEG and administration of probiotics (10^7^ cfu in 200 μl) *via* oral gavage/day, on every alternate day till 2 weeks (Abx probiotic mice). Group IV = Abx mice fed with 5% butyrate fortified chow diet (w/w) from day 7 to day 21 (Abx butyrate mice) **(A)**. Blood, liver tissue, and fecal samples were collected on day 21. The relative abundance of the *butCoAT* gene in the feces measured by qPCR and normalized to 16S rRNA bacterial gene abundance **(B)**. Fecal butyrate (expressed in µg/g of feces) on day 21 estimated by LC-MS **(C)**. The serum cholesterol in mg/dl **(D)** and hepatic 903 cholesterol expressed in µg/g liver tissue **(E)**. Expression of hepatic AUF1^p40^, Dicer1, and miR122 904 determined by qPCR **(F)**. *N* = 5/group; data are presented as mean ±SE. The experiment was repeated twice. **** represents p<0.0001, *** represents *p* < 0.001, ** represents *p* < 0.01, * represents *p* < 0.05, and ns represents not significant.

To show that the gut-derived butyrate is intimately linked with cholesterol metabolism in mice, we followed a similar approach (Wang et al., 2021) and depleted the gut microbiota of mice by daily gavage supplemented with a cocktail of antibiotics as a surrogate of germ-free mice. The treatment protocol of each group of mice is schematically presented in **Figure 6A**. The mice were divided into four groups: Group I: fed with chow diet only (untreated mice), Group II: received cocktail antibiotic (Abx mice), Group III: treated with the cocktail antibiotic for 7 days followed by probiotic treatment every other day for 21 days (Abx probiotic mice), and Group IV: Abx mice receiving butyrate from day 8 to 21 (Abx butyrate mice). We determined the relative abundance of the terminal enzyme [butyryl-CoA: acetate CoA-transferase (*butCoAT*)] of the butyrate synthesis pathway from fecal DNA samples as an indirect indicator of the relative abundance of butyrate-producing bacteria. Abx mice showed a ~100-fold decrease in the relative abundance of *butCoAT* gene compared to untreated mice which was restored to essentially a normal level in Abx probiotic mice (**Figure 6B**). To show that a decrease in relative abundance of *butCoAT* gene was faithfully reflected in butyrate production, we estimated the fecal butyrate by LC-MS. The decrease in fecal butyrate was more pronounced than the *butCoAT* gene abundance in Abx mice. There was a ~9,000-fold reduction in fecal butyrate in Abx mice compared to untreated mice, whereas with probiotic treatment, the fecal butyrate increased 70,000-fold higher compared to Abx mice and ~7-fold higher than untreated mice (**Figure 6C**). We measured serum and liver cholesterol and showed that the serum cholesterol was increased 2.5-fold on day 7 and remained the same on day 21 in the Abx mice compared to untreated mice (**Figure S8**). In Abx probiotic mice and Abx butyrate mice, the hepatic and serum cholesterol decreased significantly compared to Abx mice and returned to normal levels (**Figures 6D, E**).

The rise in serum cholesterol and decrease in fecal butyrate production were correlated with a ~1.7-fold decrease in AUF-1 and a concomitant 2-fold and 4-fold increase in Dicer-1 and mir-122, respectively, in Abx mice, compared to untreated mice. As expected, there was a significant increase in AUF-1 and decrease in Dicer-1 and mir-122 in Abx probiotic mice and Abx butyrate mice, compared to Abx mice (**Figure 6F**).

### mir-122 overexpression rescues hypocholesterolemic effect of butyrate in mice

**Figure 7:**
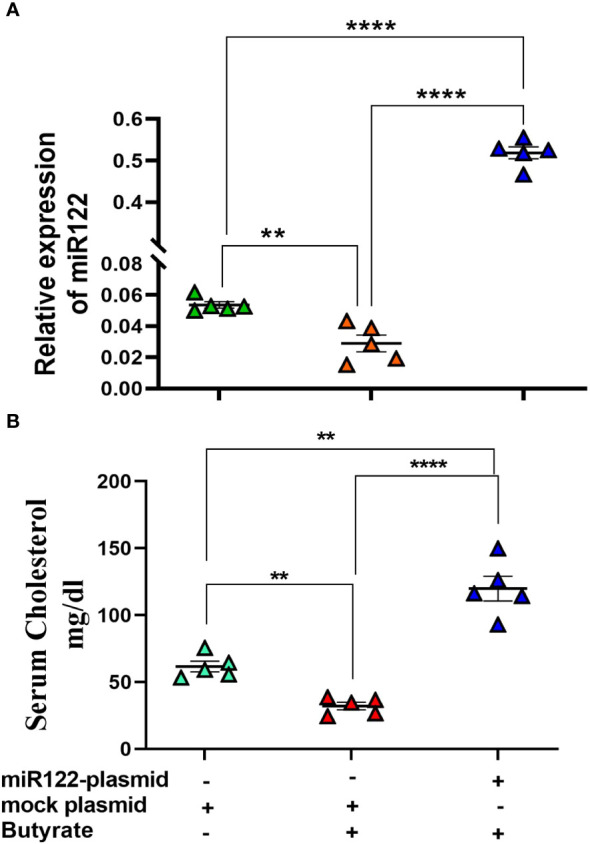
miR122 overexpression in liver rescues butyrate-induced decrease in serum cholesterol. Each butyrate-treated female mice were injected with either 25 µg in 100 µl of miR122 expressing plasmid or 25 µg in 100 µl of mock plasmid in tail vein. The mice were sacrificed 4 days post-injection. The serum cholesterol and hepatic miR122 expression was measured. *N* = 5; the data are presented as mean ± SE. The experiment was repeated twice. **** represents *p* < 0.0001 and ** represents *p* < 0.01.

From the previous experiments, it was apparent that butyrate regulates cholesterol biogenesis by exploiting mir-122. Therefore, we validated our observation by overexpressing mir-122 in butyrate-treated mice for which plasmid expressing mir-122 was injected through the tail vein of mice fed with chow diet supplemented with butyrate. Another group of mice were injected with mock plasmid, and on day 4 post-injection, the animals were sacrificed, following which serum and liver cholesterol were analyzed. We observed a 25-fold increase in mir-122 expression in the liver coupled with a 4-fold increase in serum cholesterol compared to butyrate-fed mock-injected mice (**Figures 7A, B**).

### Effect of AUF-1 knockdown by morpholino oligomer (GMO–PMO) on the expression of Dicer-1, mir-122, and cholesterol metabolic enzymes and on serum cholesterol levels

**Figure 8:**
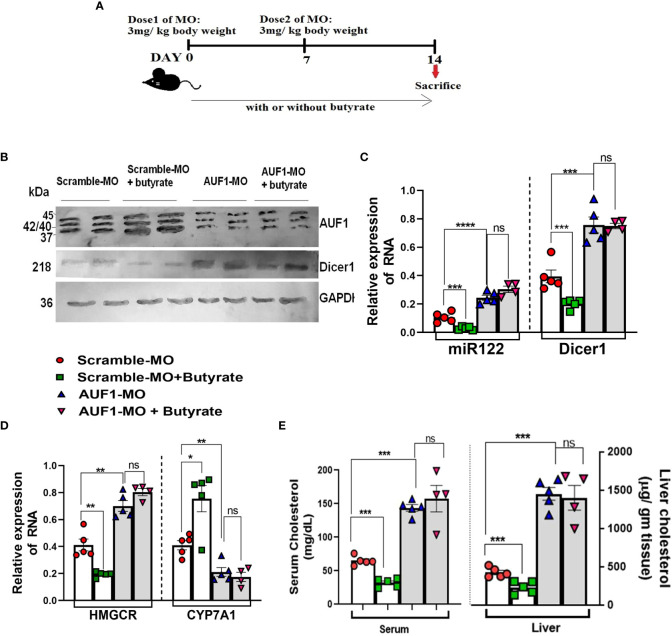
*In vivo* knockdown of AUF1 increases cholesterol synthesis by exploiting the AUF1–Dicer1–miR122–cholesterol pathway regardless of butyrate treatment. Female mice (20 in number) were divided randomly into four groups: AUF1-MO-injected mice (AUF1-MO mice), AUF1-MO-injected mice fed with butyrate supplement (butyrate AUF1 MO mice), scramble-MO-injected mice (scramble MO mice), and scramble-MO-injected mice fed with butyrate supplement (butyrate scramble MO mice); the scheme was shown schematically **(A)**. Western blots to determine hepatic expression of AUF1 and Dicer1 **(B)**. GAPDH was used as control. AP-conjugated secondary antibodies were used in Western blot. Expression of hepatic miR122 and Dicer1 determined by qPCR **(C)**. Hepatic expression of cholesterol metabolizing enzymes: HMGCR and Cyp7A1 determined by qPCR **(D)**. Serum and liver cholesterol were measured and expressed in mg/dl and µg/g tissue, respectively **(E)**. *N* = 5/group; data are presented as mean ±SE. The experiment was repeated twice. **** represents *p* < 0.0001, ***represents, ** represents p<0.01, * represents p<0.05 *p* < 0.001, and ns represents not significant.

To establish the basic tenet that AUF-1 is an important factor in the butyrate-mediated cholesterol homoeostasis, we opted for AUF-1 knockdown (AUF-1-KD) in mice using a novel cell-penetrating morpholino oligomer (GMO–PMO). In this study, GMO–PMO specific to AUF-1 was denoted as AUF-1-MO and the corresponding scramble one was denoted as scramble-MO. Mice that received morpholino on days 0 and 7 were sacrificed on day 14, as depicted pictorially (**Figure 8A**). We showed that there was no difference in AUF-1 expression between normal and scramble-MO-treated mice showing that the scramble-MO did not interfere in AUF-1 expression though the AUF-1-MO did, supporting that the effects of the AUF-1 and scramble GMO–PMOs were sequence specific (**Figures S10A, B**), and in subsequent experiments, normal mice were not included. The results showed that AUF-1-MO significantly downregulated the AUF-1 isoform not only in the liver (**Figure S10A**), but also in other organs such as the kidneys and heart (**Figure S10C**). Furthermore, butyrate treatment in the scramble-MO or AUF-1 MO mice did not change their respective AUF-1 status in liver (**Figure 8B**). AUF-1-KD in hepatic mir-122 and Dicer-1 expression was studied, and it was observed that compared to scramble-MO alone, the combination of butyrate with scramble-MO led to a significant downregulation of mir-122 and Dicer-1. As expected, AUF-1-MO treatment in mice led to upregulation of both mir-122 and Dicer-1 and remained unaltered when combined with butyrate treatment (**Figure 8C**). We also studied the impact of upregulation of mir-122 in AUF-1-KD mice for which two genes of cholesterol metabolic enzymes, HMGCR and CYP7A1, are considered of cholesterol homeostatic pathway and are the known targets of mir-122. As expected, in the scramble-MO group receiving butyrate, there was downregulation of *hmgcr* coupled with upregulation of *cyp7A1*. In AUF-1-KO mice, regardless of butyrate treatment, there was a significant upregulation of *hmgcr* coupled with downregulation of *cyp7A1* (**Figure 8D**). Both mir-122 and Dicer-1 capture a reciprocal relation between *hmgcr* and *cyp7A1* (**Figure 8E**). As expected, butyrate treatment of scramble-MO mice showed a significant decrease in serum and hepatic cholesterol, whereas AUF-1-MO-KD mice showed an amply surged cholesterol level, which remained unaltered regardless of butyrate treatment (**Figure 8E**).

## Discussion

This discourse attempts to shed light on a deeper understanding of the role of butyrate in host cholesterol homeostasis for which the following questions were asked: (a) What are the target genes and the intracellular players to link the effect of butyrate on cholesterol metabolism? (b) Does endogenous gut-derived butyrate exploit a similar molecular pathway like exogenously fed butyrate? (c) What are the cell line and mouse phenotypes when selective important players were either overexpressed like mir-122 or knocked down like AUF-1 to bring strength to our notion?

We showed that unlike acetate and propionate, butyrate has emerged as a hypocholesterolemic short-chain fatty acid in primary hepatocytes, Huh7 cells, and HFD mice, which has warranted a mechanistic look to understand how butyrate influences a diverse repertoire of functions. To address the first point, we sought assistance of the published microarray datasets (GSE4410 and GSE45220) and showed that the key cholesterol-metabolic genes, *hmgcr*, *hmgcs1, acat2, dhcr7*, and *dhcr24*, were downregulated upon butyrate treatment, which we have also validated by qPCR in HFD mice and HFD butyrate mice except dhcr24, as it is involved in the later stages of cholesterol biosynthesis. The enzymes HMGCR and HMGCS are rate-limiting (Vock et al., 2008); DHCR7 is the terminal enzyme in the cholesterol synthesis pathway (Luu et al., 2015), whereas ACAT2 is involved in cholesterol ester formation (Anderson et al., 1998).

In addition, we also studied the expression of two other important entities: (a) *cyp7A1*, a member of the monooxygenase cytochrome P450 superfamily that catalyzes cholesterol to bile acid (Li et al., 2013), and (b) ABCA1, involved in cholesterol efflux (Yancey et al., 2003). We report the downregulation of ABCA1 expression in HFD mice compared to chow mice, which is in contrast to a previously published result (Jeon et al., 2015). The diet composition and duration of treatment of HFD may possibly explain this disparity. The expression of *acat2* and *cyp7a1* appeared to be quite opposite in HFD mice as compared to HFD butyrate mice, which may have contributed to the “lipid droplet” formation, and butyrate treatment may have caused their dissolution. HFD mice showed elevated ALT and AST, indicating hepatic stress, which returned to normal levels in HFD butyrate mice. Our results are concordant with earlier reports on the beneficial effect of butyrate on liver damage and dyslipidemia (Endo et al., 2013; Zhao et al., 2021).

It has been reported that butyrate induces profound changes in miRNA in MDCK cells (Bishop et al., 2017). We identified few important miRNA, particularly those that are associated with cholesterol metabolism, such as mir-122, miR27a, and miR27b (Krutzfeldt et al., 2005; Goedeke et al., 2015). An elegant study by others shows that the treatment of mice with antagomir-mir-122 led to the downregulation of a large number of genes, of which the top-ranking functional category includes the cholesterol biosynthesis genes, namely, *hmgcr, hmgcs1*, and *dhcr7* (Krutzfeldt et al., 2005). Additionally, mir-122 destabilizes cyp7A1mRNA by binding to its 3’-UTR (Song et al., 2010). Reports show that silencing of mir-122 in African green monkeys and chimpanzees has resulted in substantial reduction in total plasma cholesterol (Zhou et al., 2019), while in the Huh7 cell line, it rescues excess lipid deposition (Long et al., 2019).

To witness the effect of butyrate on the cholesterol metabolic landscape, we undertook studies with the Huh7 cell line, which is known to have higher mir-122 expression (Fukuhara et al., 2012). As it is well known that the function of isolated primary hepatocytes is hard to maintain, when cultured *in vitro*, these cells readily undergo dedifferentiation, causing them to lose hepatocyte function (Hu and Li, 2015); thus, Huh7 cells that mimic the function of liver were used. For *in vitro* experiments on a cancer cell like Huh7, a relatively higher dose of butyrate was needed for gene expression study. As an epigenetic modifier, butyrate may be differentially utilized in normal and cancerous cells (Donohoe et al., 2012); therefore, it is tempting to speculate that a higher dose of butyrate is required to induce an effect in Huh7. The butyrate concentration used was in tune with an earlier study (Zhao et al., 2021). Butyrate treatment showed an increase in ABCA1 expression whose function was validated by showing an increase in cholesterol efflux and a decrease in miR27a, which caused decay of ABCA1 mRNA (Goedeke et al., 2015). To link butyrate with mir-122 biogenesis, butyrate-treated Huh7 cells showed reduced Dicer-1 expression, which halted maturation of mir-122 from its precursor. The effect was butyrate-specific, because neither propionate nor acetate caused any effect.

Since Dicer-1 is an important protein for the canonical miRNA biogenesis, the question arises whether all microRNA synthesis is reduced due to Dicer-1 downregulation by butyrate. We show that the expression of miRNAs that decay ABCA1 mRNA by binding to its 3’-UTR (Goedeke et al., 2015), like miR27a but not miR27b, was inhibited due to butyrate treatment, suggesting that all miRNAs were not equally affected by butyrate treatment as there are reports of Dicer-1-independent miRNA biogenesis (Cheloufi et al., 2010; Yang et al., 2012; Kim et al., 2016) where the slicer activity of Argonaute2 (Ago2) plays a vital role in pre-miRNA cleavage (Cheloufi et al., 2010).

Dicer is critical for most miRNAs, but the 5p miRNAs appear to be produced to an extent without Dicer (Kim et al., 2016). Therefore, it will be interesting to investigate further if butyrate enhances the Dicer-independent pathway for miRNA biogenesis. Our preliminary observation of increased Ago2 expression in butyrate-treated cells (data not shown) is indicative of such phenomena. It is tempting to speculate that miR27b, which remains unaltered in butyrate treatment, may be synthesized in an Ago2-dependent manner as miR27b is found to be closely associated with Ago2 (Miao et al., 2018).

Cellular steady-state mRNA levels are maintained by the result of two opposite mechanisms, i.e., transcription and decay. There is a report that AUF-1, an RNA binding protein, lowers the Dicer-1 mRNA stability by binding to its 3’-UTR (Abdelmohsen et al., 2012). The decay of transcripts containing AU-rich elements (AREs) is another example of RNA regulation that coordinates several physiological processes through posttranscriptional regulation (Schoenberg and Maquat, 2012). AUF-1 has a multitude of functions, such as DNA binding (Enokizono et al., 2005), RNA turnover (DeMaria and Brewer, 1996), and mRNA translation efficiency (Liao et al., 2007). The AUF-1 family contains four isoforms, AUF-1^p45^, AUF-1^p42^, AUF-1^p40^, and AUF-1^p37^, and their relative levels, rather than the absolute amount of individual AUF-1 isoforms, determine the net mRNA stability of ARE-containing transcripts (Kishor et al., 2013). Although different AUF-1 isoforms have different binding affinity to specific mRNA 3’-UTRs (Kajita et al., 1995; Wagner et al., 1998), the detailed mechanism by which AUF-1 isoforms selectively regulate Dicer-1-mRNA turnover is unclear.

We showed that butyrate induces upregulation of both AUF-1^p40^ and AUF-1^p37^, but not AUF-1^p42^ and AUF-1^p45^, in Huh7 cells. Since AUF-1^p40^ expression was quite prominent among all the isoforms, the rest of the studies were done with the AUF-1^p40^ isoform only. However, it does not exclude the possibility of the role of AUF-1^p37^ in the regulatory processes. There is a previous report stating that butyrate influences alternative splicing of many proteins like vascular endothelial growth factor (VEGF), IL-18, Defensin β-1 gene, and some others (Wu et al., 2012). Therefore, one may speculate that butyrate, by influencing alternative splicing of AUF-1, may regulate gene expression. For functional validation of butyrate-induced upregulation of AUF-1, we studied hepatic expression of a known classic target of AUF-1 sphingosine kinase1 (spkh1) (Sobue et al., 2008), responsible for cellular proliferation (Sobue et al., 2008) and a prognostic marker for hepatocellular carcinoma (HCC) (Cai et al., 2017). Butyrate-mediated decay of Sphk1 may also explain anticancer effects of butyrate on HCC (Wang et al., 2013). The precise mechanism by which butyrate activates AUF-1 is not clear. A previous study showed that HDAC inhibitor promotes transcription of RNA binding protein (RBP) through the activation of the transcription factors of early growth response protein (Sobolewski et al., 2015).

Butyrate being an HDAC inhibitor (Davie, 2003) may also activate AUF-1 through these transcription factors. We showed that silencing of AUF-1 by siRNA led to the downregulation of all the isoforms of AUF-1, coupled with an increase in Dicer-1, mir-122, and cellular cholesterol, regardless of absence or presence of butyrate. Thus, butyrate may serve as a master regulator of Dicer-1 stability through AUF-1. The limitation of our study is that the commercially available siRNA appears to downregulate all the isoforms of AUF-1, which makes it is difficult for one to ascertain the importance of any specific isoform in the process. The above findings portray the sequential interplay of several intracellular players that operate seamlessly as “butyrate–AUF-1–Dicer-1–miRNA122–cholesterol–metabolic enzymes–cholesterol level”, whose elegance was further verified using the HFD-induced gut dysbiosis model. A recent study demonstrated that butyrate decreases lipid profile including cholesterol largely by the LKB1–AMK–Insig signaling pathway (Zhao et al., 2021). Interestingly, mir-122 knockdown in mice showed activation of the LKB1–AMK pathway (Long et al., 2019). This study lends credence to the notion that the LKB1–AMK–Insig pathway connects butyrate and cholesterol convergence in the downstream of the “AUF-1–Dicer-1–miRNA122” axis.

To address the second query and to show that gut-derived butyrate is the prime regulator of cholesterol homeostasis, we depleted gut bacteria by antibiotic treatment as a surrogate of germ-free mice (Kennedy et al., 2018) and then reconstituted with probiotics. The probiotic consisted of *Clostridium butyricum*, lactic acid-producing bacteria (LAB), *Bacillus mesentericus*, and *Streptococcus faecalis*, which produces SCFA either directly or indirectly (He et al., 2005; Bourriaud et al., 2005; Isono et al., 2007; Mishra et al., 2019).

The final step of butyrate production is usually catalyzed by *butCoAT* and butyrate kinase (*buk*) (Louis and Flint, 2007). In a metagenome-based study, it was predicted that the *butCoAT*-mediated route is 10-fold more abundant than that mediated by *buk* (Louis et al., 2004). In our investigation, we have studied the butyrate production indirectly by measuring the relative abundance of *butCoAT* gene in the fecal samples and directly by measuring the fecal butyrate concentration by LC-MS. Oddly enough, we observed that the fecal butyrate concentration was much higher as compared to *butCoAT* gene abundance in the probiotic-treated group; the cause of such temporal mismatch is not clear. Usually, the acetyl–CoA pathway contributes to 79.7% of all butyrate production in gut microbes (Vital et al., 2014). However, other pathways like lysine, glutarate, and 4-aminobutyrate pathways also contribute to butyrate production (Vital et al., 2014). These pathways are prevalent in Firmicutes and some other phyla, such as Fusobacteria and Bacteroidetes (Barker et al., 1982. Buckel and Barker, 1974),. It is tempting to speculate that other butyrate-producing pathways may operate to contribute to the total butyrate pool. Harmonious to our result of increased serum and liver cholesterol in Abx mice, an earlier report showed that depleting intestinal microbiota by antibiotics enhanced bile acid absorption and expression of HMGCR and HMGCS in liver, resulting in 55% increase in serum cholesterol (Le Roy et al., 2019).

Another study showed that germ-free mice display 1.5 times higher liver cholesterol than conventional mice (Mistry et al., 2017). To address the third point, we showed that mir-122 is indeed involved in the recovery of serum cholesterol, and we overexpressed mir-122 in butyrate-treated mice, which showed appreciable recovery of serum cholesterol. To further establish the link between AUF-1 and cholesterol homeostasis, we knocked down AUF-1 by deploying morpholino oligomers (MO), which are short single-stranded DNA analogues that are built upon a backbone of morpholine rings and are resistant to host enzymes present, a characteristic that makes them highly suitable for *in vivo* applications. In MO, the guanidinium units that are tethered with the antisense are more rigid, which helps to get a favorable conformation to bind with the complementary mRNA (Corey and Abrams, 2001). The lower molecular weight of MO helps in endosomal escape efficiency (Abes et al., 2008). Notably, morpholino-based therapy for Duchenne muscular dystrophy (DMD) has been approved by the FDA, which is now a hallmark for morpholino-based antisense therapy (Bertoni, 2014). The chimera of GMO- and PMO-producing MO used in this investigation bears a unique proof of self-transfecting ability and nonuse of vehicle for delivery, eliminating the possibility of vehicle-induced toxicity (doi: https://doi.org/10.1101/2021.06.04.447039). Since AUF-1 knockout mice have high mortality due to high septicemia (Lu et al., 2006), the selective AUF-1 knockdown (KD) by MO offered a unique platform to test our hypothesis *in vivo*. The rise in serum cholesterol along with the increase in *hmgcr* and the decrease in *cyp7A1* expression in the liver of AUF-1 KD mice compared to scramble was noted, indicating that AUF-1 is the master regulator of cholesterol biogenesis. Furthermore, it was also observed that exogenous butyrate fails to correct hypercholesterolemia in AUF-1 KD mice. Importantly, AUF-1 KD showed high levels of Dicer-1 and mir-122 expression compared to scramble. The increase in mir-122 and the decrease in *cyp7A1* in AUF-1-MO mice is significant as mir-122 directly binds to cyp7A1 mRNA (Song et al., 2010) leading to its decay.

Overall, the present study demonstrates the effectiveness of butyrate as a powerful regulator of cholesterol homeostasis at multiple layers. By aligning a series of *in vitro* and *in vivo* experiments, we report how the intestinal microbial butyrate regulates cholesterol balance by involving intercellular players in tandem as follows: “butyrate–AUF-1–Dicer-1–mir-122–cholesterol metabolic enzymes–cholesterol level” where the individual members contribute either *via* up- or downregulation.

## Materials and methods

### Propagation of Huh7 cells and estimation of cellular protein and cholesterol

Huh7 cells were cultured in DMEM supplemented with 10% FCS and 50 µg/ml gentamycin at 37°C with 5% CO2. Cellular viability was assayed by MTT. Cholesterol estimation was performed using Amplex Red Cholesterol Assay kit. An aliquot of cell lysate was used for protein estimation using the Pierce™ BCA Protein Assay Kit following manufacturer’s protocol.

### Mice and animal ethics

Pathogen-free C57BL/6 mice (6 weeks old) were procured from the institutional animal facility of Indian Council of Medical Research-NICED, Kolkata, India. All the study protocols were approved by the Institutional Animal Ethics Committee of Indian Council of Medical Research-NICED, Kolkata, India (NICED/BS/MB-001/2019). All mice were housed under a 12-h light/dark cycle at a controlled temperature. Experiments have been carried out in accordance with the guidelines laid down by the Committee for the Purpose of Control and Supervision of Experiments on Animals (CPCSEA), Ministry of Environment and Forests, Government of India, New Delhi, India.

### Dietary supplementation of sodium butyrate

The dietary supplementation studies were performed as reported earlier with minor modifications (Xu et al., 2018). Briefly, a group of 10 adult C57BL/6 mice (with 5 in each group) were fed with HFD having 20% kcal protein, 20% kcal carbohydrate, 60% kcal fat, and trace amount of cholesterol (0.007% w/w). After 4 weeks of HFD feed, five animals from the group were selected randomly and was further fed with 5% sodium butyrate (w/w) (sodium butyrate in solid form was thoroughly blended into HFD) for 15 days (HFD butyrate mice). The remaining mice were continued with HFD for 15 days (HFD mice). Fresh diet was replenished in alternate days. Age-matched mice fed with regular chow diet served as the normal group (chow mice).

### Antibiotic treatment

The endogenous intestinal microbiota of 4-week-old chow-fed mice was depleted by gavage with broad-spectrum antibiotics over 7 days (Reikvam et al., 2011). The antibiotics solution consisted of ampicillin, metronidazole, and vancomycin diluted in sterile water. Mice received 200 mg/kg of ampicillin and metronidazole and 100 mg/kg of vancomycin once a day. Henceforth, the antibiotic-treated mice are called Abx mice.

### Probiotic treatment

Probiotic (each capsule containing 50 million lactic acid bacteria, 2 million *C. butyricum*, 30 million *S. faecalis*, and 1 million *B. mesentericus*) treatment was done as described previously with minor modifications (Le Roy et al., 2018). Briefly, a group of 20 mice (4 mice in each cage) were fed with cocktail antibiotic for 7 days and 5 mice were subjected to bowel cleansing with 1.2 ml of polyethylene glycol (PEG) solution for each mouse prior to probiotic administration. Since mice are coprophagous, they were placed in clean cages having wired net at the bottom of the cage to prevent feeding of feces. Feces and blood (from tail vein) were collected before bowel cleansing.

The PEG solution contained PEG 3350 (77.5 g/L), sodium chloride (1.9 g/L), sodium sulfate (7.4 g/L), potassium chloride (0.98 g/L), and sodium bicarbonate (2.2 g/L) diluted in sterile tap water and was divided in five equal doses (200 µl/mouse/dose) that were administered by oral gavage at 30-min intervals after a 2-h fasting (free access to water). The PEG solution and the inoculum were provided 24 h after the last antibiotic gavage. Each capsule of probiotic was suspended in 1 ml of water and two inoculations of probiotics (200 μl) were administered *via* oral gavage to Abx mice, every other day till 2 weeks. The mice were transferred to a sterile cage and straw. All animals received autoclaved deionized water and chow diet *ad libitum* for the next 14 days. A group of five Abx mice were continued with antibiotic feeding along with 5% sodium butyrate supplemented with chow diet for next 14 days (Abx butyrate mice). Another group of five Abx mice were continued with antibiotic treatment for the next 14 days. Age-matched mice (five in number) fed with normal chow diet without antibiotic treatment throughout the period of 21 days served as a control (chow mice).

### mir-122 overexpression in mice

mir-122 was overexpressed in mouse liver as described earlier (Chang et al., 2004; Ghosh et al., 2013). The mir-122 expression plasmid or empty plasmid (mock) was injected through the tail vein of butyrate-treated mice at a dose of 25 µg of DNA in 100 µl of saline/mouse. Mice were sacrificed on day 4 post-injection, and serum cholesterol and mir-122 expression in liver were measured.

### Mice fecal sample collection

Fresh fecal samples of all mice were collected at a fixed time of a day to minimize possible circadian effects. Samples were collected into empty sterile microtubes on ice and stored at −80°C within 1 h for future use.

### Fecal DNA extraction and determination of butyryl-coenzyme A (CoA):acetate CoA-transferase (ButCoAT) gene abundance by qPCR

The QIAamp DNA stool minikit (Qiagen) was used to extract DNA in QIAcube (Qiagen) from 40 mg of fecal pellet from each mouse. The DNA was measured using a QIAxpert spectrophotometer (Qiagen). To compare the relative abundance of bacteria having the *butCoAT* gene in fecal DNA of mice, qPCR analysis was carried out and quantified by SYBR green and the data were normalized to total bacterial abundance using 16S rRNA-bacterial primers (Berding and Donovan, 2018; Jian et al., 2020). The sequence of the primers used is listed in **Table S1**.

### Measurement of butyrate in feces by LC-MS

The fecal content of each mouse (50 mg wet weight) was dissolved in distilled water at 1:10 (w/v) and homogenized in a Dounce homogenizer. Then, the content was centrifuged at 10,000*g* for 10 m and the supernatant was filtered through a 0.45-µm syringe filter. The supernatant was further diluted in LC-MS grade distilled water up to a final volume of 1 ml and was subjected to LC-MS analysis for SCFA as described by Cheng et al. (2020). In brief, a calibration curve was generated by using varying calibrations of internal standards of butyrate from 2 to 50 µmol. The LCMS separation was performed on the Agilent 1290 Infinity LC system, which was coupled to Agilent 6545 Accurate-Mass Quadrupole Time-of-Flight (QTOF) with an Agilent Jet Stream Thermal Gradient Technology with electrospray ionization (ESI) source. The suitable MS parameters were optimized and the high-resolution mass spectra were obtained by performing the analysis in negative ionization mode. The chromatographic separation was achieved on an Agilent ZORBAX SB-C18 column (2.1 × 100 mm, 1.8 µm) as a stationary phase. The mobile phase consisted of a linear gradient of 0.1% (v/v) aqueous formic acid (A) and methanol (B): 0–1.0 min, 0% B (v/v); 1.0–3.0 min, 0%–30% B (v/v); 3.0–6.0 min, 30%–100% B (v/v); 6.0–12.0 min, 100% B (v/v); 12.50–15.0 min, 0% B. The column was reconditioned for 5 min before the next injection. A flow rate of 0.2 ml/min and a varying injection volume were used for analysis. The UPLC system was assembled with a diode array detector (DAD) and an auto sampler. The peak area of each SCFA was used to calculate the amount of SCFA present, which was further normalized by the injection volume. The data were represented as the amount of butyrate present per gram of feces.

### Preparation of liposome and loading of cholesterol to Huh7 cells

Liposomes were prepared using 22-NBD-cholesterol [22-(N-(7-nitrobenz-2-oxa-1,3-diazol-4-yl)amino)-23,24-bisnor-5-cholen-3β-ol] as previously described (Kheirolomoom and Ferrara, 2007). Briefly, 22-NBD-cholesterol (1 mg), cholesterol (4.8 mg), and phosphatidylcholine (8 mg) were dissolved in 1 ml of chloroform. The solvent was evaporated to obtain a homogeneous thin lipid film and put into a 100-ml round-bottom flask, followed by solvent removal by rotary evaporation under reduced pressure. The lipid film was then hydrated in serum-free DMEM and sonicated (Microson Ultrasonic cell disruptor with a Misonix 2-mm probe) at 4°C three times for 1 min each at maximum output and filtered with a 0.2-µm Millipore filter (Banerjee et al., 2009). Huh7 cells were plated in a 96-well plate at a cell density of 10^5^cells/well. Cells were equilibrated with 22-NBD-cholesterol-liposome overnight. After incubation with liposome, cells were washed with 1× PBS and were subjected to treatment with or without butyrate.

### Cholesterol efflux assay

The cholesterol efflux assay was performed as previously described (Low et al., 2012). Briefly, cholesterol-loaded cells were washed with 1× PBS and then treated with butyrate for the next 24 h. Following treatment with or without butyrate, cells were washed with 1× PBS and incubated in serum-free DMEM for the next 18 h. After the specified time of incubation, cells were treated with 1, 5, and 20 µg/ml HDL, respectively, for 4 h to induce efflux. Thereafter, cell supernatant was collected and cells were lysed using methanol. The fluorescence intensity (FI) of 22-NBD-cholesterol in the medium and cell lysate was detected by an MT-600F fluorescence microplate reader (Corona Electric, Hitachinaka, Japan) using 469-nm excitation and 537-nm emission filters in a black polystyrene 96-well plate (Costar, Corning Incorporated, USA). The percent cholesterol efflux was calculated as follows: % cholesterol efflux = (FI medium × 100)/(FI medium + FI lysate).

### GMO–PMO treatment to knock down AUF-1

The GMO–PMO was dissolved in saline to make a concentration of 1 mM, which was equivalent to 8.2 mg/ml. *In vivo* knockdown of AUF-1 was carried out in mice by injecting AUF-1 GMO–PMO (AUF-1-MO) through the tail vein on day 1 and day 7 at a dose of 3 mg/kg body weight. The effective dose was previously determined based on AUF-1 expression in liver by a pilot study. Another group of AUF-1-MO-injected mice were fed with 5% butyrate-supplemented chow diet from day 1 and continued till day 14. Blood was collected from the tail vein on day 7 and day 14 for cholesterol estimation. Mock GMO–PMO-treated animals are called scramble-MO controls. Another group of scramble-MO were fed with butyrate for 14 days after the first dose injection. Animals were sacrificed after day 14 and tissue was collected for further analysis.

### Statistical analysis

All values in the figures and text are expressed as arithmetic mean ± SEM. Data were analyzed with GraphPad Prism Version 8.01 software and statistical significance between groups was determined using unpaired Student’s *t*-test. *p* values <0.05 were considered statistically significant. In the experiment involving Western blot, the figures shown are representative of at least three experiments performed on different days.

## Data availability statement

The original contributions presented in the study are included in the article/**Supplementary Material**. Further inquiries can be directed to the corresponding author.

## Ethics statement

The animal study was reviewed and approved by Institutional Animal Ethics Committee of Indian Council of Medical Research-NICED, Kolkata, India, (NICED/BS/MB-001/2019).

## Author contributions

OD performed majority of the experiments and analyzed data. JK and AGh designed and synthesized GMO–PMOs. AGa, SG, MC, AM, SSG, and DM performed experiments. MD performed TEM study. BM and SS prepared the manuscript and analyzed data. MB conceived, designed, supervised, arranged funding, and wrote the manuscript. All authors contributed to the article and approved the submitted version.
