# Supplementary material for: AUF-1 knockdown in mice undermines gut microbial butyrate-driven hypocholesterolemia through AUF-1–Dicer-1–mir-122 hierarchy
Source: Front Cell Infect Microbiol. 2022 Dec 19;12:1011386. doi: 10.3389/fcimb.2022.1011386 (PMC9806232; doi:10.3389/fcimb.2022.1011386)
Supplement: Supplementary file 1 [file DataSheet_1.pdf]

SUPPLEMENTAL INFORMATION

Title: AUF-1 knock down in mice overarches gut microbial butyrate driven hypo-cholesterolemia by conjuring AUF-1-Dicer-1-miR122 hierarchy

Short title: Gut microbial butyrate regulates cholesterol homeostasis

Oishika Das[1], Jayanta Kundu[2], Atanu Ghosh[2], Anupam Gautam[3], Souradeepa Ghosh[4], Mainak Chakraborty[1], Aaheli Masid[1], Samiran Sona Gauri[4], Debmalya Mitra[1], Moumita Dutta[1], Budhaditya Mukherjee[4], Surajit Sinha[2], Moumita Bhaumik[1*]

Materials and Methods

Reagents and Chemicals

Chow diet (Harlan Teklad LM-485), High Fat Diet (HFD) (Harlan Teklad TD93075) was purchased from ICMR-NIN, Hyderabad, India. Dulbecco's modified Eagle's medium (DMEM), HBSS and foetal calf serum (FCS) were purchased from GIBCO (Waltham, MA, USA). Gentamycin, BCA protein assay kit, BCIP-NBT kit were purchased from Thermofisher (Waltham, MA, USA). EDTA, Collagenase IV, HEPES, Penicillin, streptomycin, ampicillin, vancomycin, metronidazole, MTT (3-(4,5-dimethylthiazol-2-yl)-2,5-diphenyl tetrazolium bromide), Triton X100, PMSF, leupeptin, glycine, acrylamide, bis-acrylamide para-formaldehyde, glutaraldehyde, sodium butyrate, sodium propionate, sodium acetate, Hoechst 33342, and cholesterol estimation kit were purchased from Sigma (St. Louis, MO, USA). AST and ALT estimation kit was purchased from Transasia Biomedicals (Mumbai, India). 22-NBD-[22-(*N*-(7-Nitrobenz-2-Oxa-1,3-Diazol-4-yl)Amino)-23,24-Bisnor-5-Cholen-3β-Ol]-cholesterol, cholesterol and phosphatidyl choline were purchased from Avanti-polar lipids (Birmingham, USA). HDL was purchased from Beacon Diagnostics Pvt Ltd (Navsari, Gujrat, India). Probiotic was purchased from Bifilac (Tablets India, Chennai, India). Poly ethylene glycol (PEG) solution was purchased from Jhaver Centre (Chennai, India). Amplex red cholesterol assay kit, Lipofectamine 2000, PVDF

membrane, Trizol, Opti-MeM, were purchased from Invitrogen (Carlsbad, CA, USA). QIAamp stool mini kit was purchased from Qiagen (Hilden, Germany). Prime script first strand cDNA synthesis kit, TB Green Premix ex-Taq (Tli RNase H+) qPCR kit were purchased from Takara (Shiga, Japan). (Ripa lysis buffer, anti-β-actin antibody (polyclonal), siAUF1, Anti-AUF1 antibody (rabbit polyclonal), anti-HMGCR antibody (polyclonal), anti-ABCA1 antibody (rabbit monoclonal) were purchased from Cell Signalling Technology (Danvers, MA, USA). Anti-ABCA-5 antibody (rabbit, polyclonal) was purchased from Abcam (Cambridge, UK). Anti-Dicer1 antibody (mouse monoclonal IgG2a) and Anti-AUF1 antibody (mouse monoclonal IgG1) was purchased from Santa-cruz Biotechnology (Dallas, Texas, USA). Anti-GAPDH antibody (rabbit polyclonal) was purchased from Bio-Bharati (Kolkata, India). All primers were purchased from IDT (Lowa, USA). Human hepato-carcinoma cell line Huh7 and pmiR122 were a kind gift from Dr. Suvendranath Bhattacharya (CSIR-IICB, India). pEGFP-3ÚTRDicer1 and pEGFP were a kind gift from Dr. Myriam Goropse, NIH, USA. pEGFP-AUF1isoforms were also gifted by Prof. Andrea Pautz, University of Mainz, Gutenberg.

In silico analysis of global microarray data

Raw microarray expression data was obtained in a CEL file format using GSEOquery package (DOI: 10.18129/B9.bioc.GEOquery) from GSE45220 (human) and GSE4410 (mice) datasets. The *GEOquery* R package parses GEO data into R data structures that can be used by other R packages. Normalization and background correction of Gene Expression Measurements was performed using oligo package's Robust Multichip Average (RMA) algorithm that attempts to remove local biases across samples in order to enable relevant differential expression testing. Variations are scanned across the samples using principal component analysis and Hieracrchial clustering method. Following which, differential gene analysis was done using Limma package. The *limma* (Linear Models for Microarray

Analysis) R package has evolved as the most widely used statistical tests for identifying differentially expressed genes using Limma based adjusted P-value. P-value of <0.05 are selected. Results are annotated using library hugene10sttranscriptcluster.db for GSE45220, and huex10sttranscriptcluster.db for GSE4410. In case of genes with two or more probes aligned/mapped, only the most significant ones were chosen and the list of upregulated and downregulated genes are prepared accordingly. Genes with the smallest adjusted P-value and highest logFC values are the most significant. Amongst them genes which have a possible role in cholesterol metabolism were selected using KEGG pathway, BioGPS and Reactome databases and were sorted accordingly. All the cholesterol genes showed a downregulated expression. The data were represented as Volcano plot.

Isolation for Murine Hepatocytes:

Murine hepatocytes were isolated via Percoll method (1) with slight modifications. Briefly, euthanized C57BL/6 mice were placed on top of tissue paper pillow and incision was done to open the abdomen properly. A small incision was done to insert cannula into the portal vein and liver was perfused with 10 ml of perfusion buffer (25mM HEPES and 0.5M EDTA in HBSS without Ca^{2+} and Mg^{2+}). Digestion buffer (25mM HEPES and 25 µg/ml Collagenase IV in HBSS with Ca^{2+} and Mg^{2+}) was passed through the vena cava. Liver was carefully removed into the petridish containing digestion buffer and dissociated carefully. Cells were filtered through 100µm cell strainer (BD) and centrifuged for 5 minute at 50g. Supernatant was discarded and pellet was collected, to which 10ml of plating media (DMEM+10%FBS) was added. Subsequently, 90% of Percoll was laid over this solution and centrifuged for 10 minutes at 300g at 4°C. Supernatant was discarded and cells were washed and suspended in 10ml of DMEM.

Transmission Electron Microscopy

Liver tissue was fixed with 3% glutaraldehyde in 0.1M sodium cacodylate buffer. Subsequently, a secondary fixation was conducted with 1% Osmium tetroxide, followed by dehydration with ascending grades of acetone, and finally embedded in Agar 100 resin and

polymerization at 60°C. The ultrathin sections (40–50 nm) of the tissue were obtained using a Leica Ultracut UCT ultramicrotome (Leica Microsystems, Germany), picked up on nickel grids, and dual-stained with 2% aqueous uranyl acetate and 0.2% lead citrate. The sections were visualized under a FEI Tecnai 12 Biotwin transmission electron microscope (FEI, Hillsboro, OR, USA) at an accelerating voltage of 100 kV (2).

Food consumption

The daily consumption of food in mice was recorded every day by weighing the food given (food placed in the food receptacle at time zero) and food remaining (food left in the food receptacle 24 hours later). The cumulative food intake by each group (5 mice / group) per day was determined.

Collection of blood, preparation of serum samples, biochemical analysis, estimation of cholesterol and lipoproteins and hepatic enzymes

Blood collected from tail vein was allowed to stand for 3 h at room temperature and then serum was prepared by centrifugation at 1800 rpm analyzed for serum cholesterol by Assay Kit. Levels of aspartate aminotransferase (AST) and alanine aminotransferase (ALT) in serum were measured in using kit from Transasia Biomedicals (India).

Transfection

Cells were plated in 6-well culture plates at the density of 1×10^{6} cells/well and cultured in 2 ml serum-free medium for 24 h to 80% confluence. Transfection was performed with Lipofectamine 2000 according to the protocol recommended by the manufacturer. Briefly, 25 μl Opti-MEM medium was used to dilute 1.0 μl lipofectamine and 0.5 μg plasmid or 0.27 μg siRNA, and equal volume of the plasmid or siRNA and lipofectamine was mixed at room temperature for 15 min. Cells were transfected by adding 100 μl of the Opti-MEM medium containing lipofectamine and plasmid or siRNA at 37° C for 6 h, and then cells were grown at DMEM containing 10% FCS (3).

Fluorescence microscopy

Cells grown on glass cover slips were transfected with either pEGFP-AUF1^{p40} plasmid (4) or pEGFP-Dicer-1-3′UTR or p-EGFP (5) for 24 h. After washing with PBS, the cells were treated with 1 µg/ml Hoechst 33342 for 5 min at room temperature and washed again with PBS three times. Fluorescence images were captured with Carl Zeiss microscope equipped with a CCD camera controlled with ZEN software (Carl Zeiss, Gottingen, Germany).

Tissue homogenisation, preparation of RNA and Protein

Liver samples were dissected into small pieces and were resuspended either in RIPA Lysis buffer (20mM Tris-HCl pH 7.5, 150 mM NaCl, 1mM EDTA, 1mM EGTA, 1% NP-40, 1% Sodium deoxycholate, 2.5 mM Sodium Pyrophosphate, 1mM β-glycophosphate, 1mM Na_3VO_4, 1 µg/ml leupeptin with 1mM of PMSF immediately before use) for protein isolation or in Trizol (Invitrogen, US) for RNA isolation. The tissue was homogenized using a micropestle and centrifuged at 13,000 g for 15 min at 4°C. The clear supernatant was collected and either stored as protein lysate in -80°C or further processed to isolate RNA using the standard protocol (3).

RNA extraction and reverse transcription

Cells were cultured in 24-well plates to 80% confluence. Total RNA from cells or tissue was extracted with Trizol according to the protocol recommended by the manufacturer. The concentration of the extracted RNA was analyzed by Nanodrop spectrophotometer (Thermo) and RNA was stored at –80° C. cDNA was prepared from total RNA by reverse specific primers using Super Reverse Transcriptase MuLV Kit. The primers for the reverse transcription are listed in Table 1. U6 and GAPDH were normalized for the expressions of miRNAs and other genes of interest respectively. The total reaction volume for reverse transcription was 20 µl in which 1 µM of reverse primer, 5 ng of RNA template, 1 µl dNTP mix, 12 µl of DEPC treated water, 4 µl of 5X first strand buffer, 1 µl of 0.1 M DTT, 1 µl of

RNase inhibitor and 1 µl Super RT MuLV. Reverse transcription was carried out for 65°C for 5 minutes, followed by incubation at 55°C for 1 hour and then heat inactivating the reaction at 70°C for 15 minutes (5).

Quantitative real-time PCR

The miRNAs and mRNA levels were quantified with Applied Biosystems™ StepOne™ Real Time PCR System with RT^2 SYBR® Green qPCR Mastermix following the manufacturer's instructions. Each 20 µl qPCR reaction contained an amount of cDNA equivalent to 5 ng of total RNA, 10 µl of RT^2 SYBR® Green qPCR Mastermix, 1 µM of the forward and reverse primer (each) and nuclease free water (6). Real-time PCR was performed with the following conditions: 95°C for 10 min, 40 cycles of 95°C for 30 sec, 60°C for 1 min and 72°C for 1 min PCR product and was normalized either with GAPDH Ct value or U6 (for microRNA) as described previously (5).

The stem loop primer stock was overlaid with 100µl molecular biology grade mineral oil (Sigma). The mixture was heated to 95°C and were kept at 75°C, 68°C, 65°C, 62°C and 60°C for an hour each respectively. Thereafter, working stock of 10µM was prepared and stored in -20°C till further use (7).

Primers

Table S1: The primer sequences use for PCR amplification are as follows:

**Gene**	**Primer Sequence**	
	**Forward Primer**	**Reverse Primer**
Dicer 1(Human)	5`-GAACGCTTTTGTGCTGCTGA3`	5`CACAGGGCTCTAAAGTGGGG3`
GAPDH (Human)	5`-GAGAAGGCTGGGGCTCATTT3`	5`AGTGATGGCATGGACTGTGG3`
U6SnRNA (Human)	5`-CTCGCTTCGGCAGCACATATACT3`	5`ACGCTTCACGAATTTGCGTGTC3`
miRNA122 (Human)	5`TAGCAGAGCTGTGGAGTGTG3`	5`GCCTAGCAGTAGCTATTTAGTGTG3`
PremiRNA122	5`-CCTTAGCAGAGCTGTGGAG-3`	5`GCCTAGCAGTAGCTATTTAG-3`

(Human)		
HMGCR (Mouse)	5`-AGAGCGAGTGCATTAGCAAAG-3`	5`GATTGCCATTCCACGAGCTAT-3`
HMGCS (Mouse)	5`CTCTGTCTATGGTTCCCTGGCT3`	5`TCCAATCCTCTTCCCTGCC3`
ACAT 2 (Mouse)	5`-TTTGCTCTATGCCTGCTTC-3`	5`-GTAGAACATCCTGTCTCC-3
DHCR7 (Mouse)	5`-CACCGGCCGTGCTAGTCTGG3`	5`CAGGCTTGTAGCCCGTTCACCTC3`
CYP7A1 (Mouse)	5’-GGGCAGGCTTGGGAATTTTG -3’	5’-ACAGCTACTAGGGGGCTTCA -3’
U6snRNA(Mouse)	5-CTCGCTTCGGCAGCACATATACT-3`	5`ACGCTTCACGAATTTGCGTGTC-3`
Dicer 1 (Mouse)	5`-GTTGGTCTGAGCACTCCCTTC-3`	5`AGGCCCAGGACACTGTTTAC-3`
Auf-1p40 (Mouse)	5`-AGAACGAGGAGGATGAAGGGA-3`	5`TGTGTCTGGAGAAAGGCCAC-3`
GAPDH (Mouse)	5`-AGAGAGGCCCAGCTACTCG-3`	5`GGCACTGCACAAGAAGATGC-3`
Occludin (human)	5’-TCACTTTTCCTGCGGTGACT-3’	5’- GGGAACGTGGCCGATATAATG-3’
miRNA122(Mouse)	5`-GCTCGACCTCTCATGGGC-3`	5`TTAAGCCCTGCGTGTCCTCC-3`
PremiRNA122 (Mouse)	5`-CCTTAGCAGAGCTGTGGAG-3`	5`GCCTAGCAGTAGCTATTTAG-3`
miRNA27a (Mouse)	5-`ACACTTTCACAGTGGCTAA-3`	5-`GTGCAGGGTCCGAGGT-3`
miRNA27a SL	5-`GTCGTATCCAGTGCAGGGTCCGAGGTATTCGCACTGGATACGACGCGGAAC-3`	
ButCoAT	5’ GCIGAICATTTCACITGGAAYWSITGGCAY 3’	5’ CCTGCCTTTGCAATRTCIACRAANGC 3’
16S rRNA	5’ AGAGTTTGATCCTGGCTCAG 3’	5’AAGGAGGTGWTCCARCC 3’

Western blot

Liver tissue protein or cell lysate were extracted in RIPA Lysis buffer. Protein concentration was measured using Pierce ™ BCA Protein Assay Kit. Proteins (50 μg/lane) were separated by using SDS-PAGE on 10% gel under reducing condition and electro transferred to PVDF membrane in a transferred buffer (25mM Tris-HCl, 150mM Glycine, 20% Methanol). Membranes were blocked at room temperature with 5% non fat skim milk in TBS for 2 hours, and then incubated with primary antibody against specific protein. The membranes

were incubated either with the horseradish peroxidase (HRP)-conjugated secondary antibodies or Alkaline phosphatase (AP)-conjugated antibodies at 37° C for 1 h. For HRP-conjugated antibody treatment, SuperSignal West Pico chemiluminescent substrate kit (Thermo) was used to visualize the blotting results. The blots were imaged with Fluor Chem R system (ProteinSimple, San Jose, CA, USA) (5). For AP-conjugated antibody treatment, BCIP-NBT substrate kit (Thermo) was used to visualize the blotting results (8).

Table S2: Antibody used for Western Blots

**Name of Antigen**	**Raised in**	**Source**	**Dilutions used**
HMGCR	Rabbit	Cell Signalling Technology	1:1000
ABCA1	Rabbit	Cell Signalling Technology	1:1000
ABCA5	Rabbit	Abcam	1:1000
AUF-1 (p37 specific)	Rabbit	Cell Signalling Technology	1:1000
AUF-1/ HNRNP	Mouse	Santa Cruz	1:100
Dicer 1	Mouse	Santa Cruz technology	1:1000
β Actin	Rabbit	Cell Signalling Technology	1:1000
GAPDH	Rabbit	BioBharati	1:1000
Anti-mouse-HRP conjugated secondary antibody	Horse	Cell Signalling Technology	1:5000
Anti-rabbit- HRP conjugated secondary antibody	Goat	Cell Signalling Technology	1:5000
Anti-mouse-AP	Goat	Abcam	1:5000

conjugated secondary antibody			
Anti-rabbit-AP conjugated secondary antibody	Goat	Abcam	1:5000

Synthesis of Guanidinium Morpholino Oligonucleotides- Protected chlorophosphoramidate Morpholino Oligonucleotides (GMO-PMO)

All reagents were purchased from commercial sources and used without further purification, unless otherwise mentioned. All reactions were carried out in oven-dried glassware under argon atmosphere. Solvents were purified and dried according to recommended procedures. Thin-layer chromatography (TLC) was carried out on sheets of silica gel 60 F254 on aluminium (layer thickness 0.25 mm, Merck). Visualization of the developed chromatogram was achieved with UV light and ceric ammonium molybdate (CAM) or ninhydrin stains. Chromatographic purification of products was accomplished by column chromatography on silica gels (mesh 100-200 or 230–400). UV/Vis spectra were recorded on Agilent Cary 3500 UV Visible spectrometer. Matrix-Assisted Laser Desorption Ionization (MALDI) mass spectra were recorded on BrukerultrafleXtreme MALDI-TOF/TOF system.

Synthesis of Fmoc protected thiourea morpholino active monomer

For the synthesis of GMO (Guanidinium Morpholino Oligonucleotides, GMO) part of GMO-PMO chimera, the Fmoc protected thiourea MMTr-morpholino active monomers of C, A and G nucleosides were required. They were synthesized using the modified protocol as described earlier (8).

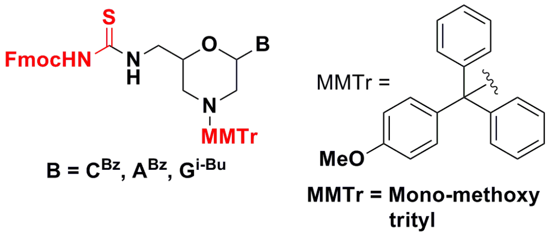

Fmoc-Thio monomer was used for the synthesis of guanidinium morpholino ligomer (GMO)

Synthesis of Trityl protected chlorophosphoramidate morpholino active monomer

For the synthesis of PMO we have used chlorophosphoramidate monomers of A, T, G and C which was synthesized as per earlier report (9).

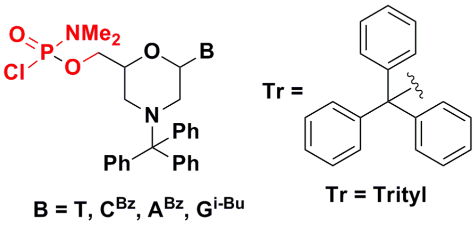

Tr-chlorophosphoramidate morpholino monomer was used for the synthesis of morpholino ligomer (MO)

Functionalization of solid support with linker and loading monomer

Prior to solid phase synthesis of GMO-PMO sequences, polystyrene solid support was functionalized with aminocaproic acid linker and loading monomer as per earlier report (10).

***Solid phase synthesis of GMO-PMO (*Fig S9 A)**

After the successful incorporation of linker and loading monomer on polystyrene solid support, coupling for GMO synthesis was initiated. For GMO synthesis 5 equivalent of Fmoc protected thiourea, 5 equivalents of $HgCl_2$ and 5 equivalent of NEM were added in NMP solvent. This step was repeated for another two times in 2 hrs interval. Total coupling time per GMO unit was 6 hrs. Excess reagents were washed with 20 % Thiophenol-NMP and NMP. Unreacted amine was capped with (1:1) mixture of 10 % Ac_2O-NMP and 10 % DIPEA-NMP. MMTr group was deprotected using the deblocking cocktail (CYPTFA) (10, 11). The synthetic cycle (washing, coupling, capping and deblocking) was repeated for another three GMO monomers (see the structures above) to get the GMO unit. The GMO pentamer was further reacted with chlorophosphoramidate monomer (see the structure above). The morpholino part was synthesized as per our reported method (11). Full length GMO-PMO was cleaved from solid support using 33 % aqueous ammonia at 55°C for 16 hrs

and purified by acetone precipitation. Purity of the synthesized GMO-PMOs were checked in HPLC (Fig S9B and C) and characterized by MALDI-TOF. Henceforth GMO-PMO will be denoted as MO.

Sequence of AUF1-MO

5´-TCCGAACTGCTCCTCCGACATAGTG-3´ targets the following AUF1 mRNA region [Red letter represents GMO part]

MALDI-TOF [M + H]$^{+}$: Mass calculated for $C_{285}H_{437}N_{142}O_{92}P_{20}$ was 7943.8907, found 7945.703.

CDS: [314:1382](+)

TACTTTGCTGCTAGTTTCGGTTCGCGGCGGCGGCGGCGTCGGCGGGTGTCG TCTTCGGCGGCGGCAGTAG[CACT(ATG)TCGGAGGAGCAGTTCGGA]GGGG

Sequence of scramble-MO

5'-TC**GC**AACT**CG**TCCTCC**AG**CATAGTG-3' [Red letter represents GMO part]

MALDI-TOF [M + H]$^{+}$: Mass calculated for $C_{285}H_{437}N_{142}O_{92}P_{20}$ was 7943.8907, found 7942.554.

FIGURE LEGENDS

Figure S1:

Cellular cholesterol status in murine primary hepatocytes and Huh7 cells before and after treatment with either butyrate or aceatate or propionate and HMGCR expression with butyrate treatment in Huh7 cells

Cellular cholesterol status in murine primary hepatocytes (A) and Huh7 cells (B) in response to increasing concentration of butyrate or propionate or aceatate expressed as µg cholesterol / mg cellular protein. Percent decrease in cholesterol in primary hepatocytes with butyrate treatment (A, inset). Percent viability of Huh7 cells at higher dose of butyrate treatment (B, Inset). Western blot of HMGCR expression as a function of butyrate concentration (B). The corresponding densitometry using ImageJ showing relative expression of HMGCR with respect to β-actin control (C). (N=3). *** represents $p<0.001$ compared to untreated control.

Figure S2

Food consumption, body weight, HDL, LDL status and hepatic enzymes in normal-mice, HFD-mice and HFD-butyrate-mice.

Percentage increase in body weight as a function of days (A) and inset showing percent increase in body weight on day 45 (A, inset), cumulative food consumption of 5 mice (in gms) / day was estimated from measuring food every 24 h over a period of 15 days in all the groups. Arrow on the abscissa indicated starting of butyrate treatment. (B), and liver enzymes (ALT & AST) expressed in

U/ml (C) were determined in all three groups of mice. N = 5 / group, data is represented as mean ±SE. The experiment was repeated thrice. ** represents $p<0.01$, * represents $p<0.05$, ns represents not significant.

Figure S3:

Volcano plot of the publicly available microarray data of control vs butyrate treated HeLa cells (GSE45220) and colon epithelial cell MCE301 (GSE4410) (A). Blue dots, red dots, grey dots represents genes that are down-regulated, up-regulated and non significant respectively. Yellow dots (encircled in red) and purple dots (encircled in light blue) represent cholesterol metabolising and other lipid metabolising genes respectively. The common important genes related to cholesterol metabolism that were down-regulated in both datasets are presented as 1-5 in the box (B).

Figure S4

Analysis of ABCA1 and ABCA5 expression in Huh7 by western blot, functional analysis of cholesterol efflux with and without butyrate treatment and status of miR122, Dicer1 and AUF1 in presence and absence of either butyrateor aceatate or propionate.

Analysis of expression of ABCA1 and ABCA5 in Huh7 cells by western blot as a function of butyrate concentration (A). The corresponding densitometry using ImageJ showing relative expression of ABCA1 and ABCA5 with respect to β-actin control (B). Percent cholesterol efflux as function of butyrate concentration in the form of 22-NBD-cholesterol was monitored by measuring 22-NBD fluorescence. Huh7 cells were loaded with liposomal 22-NBD-cholesterol for 24 h and subsequently treated with 20mM butyrate. The cells were washed and equilibrated in serum free medium for 18h. Thereafter the cells were treated with or without HDL (1 µg/ml, 5 µg/ml and 20 µg/ml). The fluorescence intensity (FI) of 22-NBD-cholesterol in the medium and cell lysate was detected by MT-600F fluorescence microplate reader (Corona Electric, Hitachinaka, Japan) using 469 nm excitation and 537 nm emission filters in a black polystyrene 96-well plate. The efflux (%) was calculated as $(FI_{sup}\ X\ 100)/\ (FI_{sup} + FI_{cell\ lysate})$ (C). Expression of pre-miR122 , miR122, Dicer1 and AUF1 (D) after butyrate or propionate or aceatate treatment 20 mM each as measured by qPCR. N ≥3 for all data sets, data is represented as mean ±SE. *** represents $p<0.001$, ** represents $p<0.01$, * represents $p<0.05$, ns represents not significant.

Figure S5

Expression Sphingosine kinase1 (Sphk1) as a function of butyrate concentration in Huh7 cells as measured by qPCR N=2. The data is represented as mean ± SE. *** represents $p<0.001$, ** represents $p<0.01$, * represents $p<0.05$.

Figure S6

Hepatic expression of miR27a and miR27b in normal-mice, HFD-mice and HFD-butyrate-mice

Hepatic expression of miR27a (A) and miR27b (B) in normal-mice, HFD-mice and HFD-butyrate-

mice measured by qPCR. N=4 /group, data is represented as mean ±SE. *** represents $p<0.001$, **represents $p<0.01$, * represents $p<0.05$.

Figure S7

Determination of fecal butyrate by LC-MS

Representative peak of butyrate in LC-MS. Standard (2 mg/ml) (A), Normal-mice (B), Abx-mice (C) and Abx-probiotic-mice (C).

Figure S8

Antibiotic treatment increases serum cholesterol

The serum cholesterol of normal-mice (A) and Abx-mice (B) on day 7 and day 21.

Figure S9

Synthesis cycle of GMO-PMO (MO) and characterization of scramble-MO and AUF1-MO by HPLC.

Synthesis cycle of GMO-PMO (MO) (A), HPLC purity peak for AUF1-MO (B) and scramble-MO (C).

Figure S10

Specificity of AUF1-MO in selective knockdown of AUF1 in mice

Mice were injected in the tail vein with either 5 mg/kg body weight or 3 mg/kg body weight of AUF1-MO/ scramble-MO. The liver samples were collected 7 days post injection and western blot analysis was performed to determine the expression of AUF1 (A) and corresponding densitometric analysis of AUF1 expression by ImageJ (B). Western blot of AUF1 in heart and kidney of mice receiving AUF1-MO (C). GAPDH was used as control for all experiment. The anti-AUF1 antibody (Cell signalling) used in this study was specific to AUF-1^{p37}. N=2

References

1. Goncalves, L. A., A. M. Vigario, and C. Penha-Goncalves. 2007. Improved isolation of murine hepatocytes for in vitro malaria liver stage studies. *Malar J* **6**: 169.
2. Eid, N., Y. Ito, K. Maemura, and Y. Otsuki. 2013. Elevated autophagic sequestration of mitochondria and lipid droplets in steatotic hepatocytes of chronic ethanol-treated rats: an immunohistochemical and electron microscopic study. *J Mol Histol* **44**: 311-326.
3. Wu, X., Y. Yang, Y. Huang, Y. Chen, T. Wang, S. Wu, L. Tong, Y. Wang, L. Lin, M. Hao, Z. H. Zhong, F. Zhang, and W. Zhao. 2018. RNA-binding protein AUF1 suppresses miR-122 biogenesis by down-regulating Dicer1 in hepatocellular carcinoma. *Oncotarget* **9**: 14815-14827.
4. Pautz, A., K. Linker, S. Altenhofer, S. Heil, N. Schmidt, J. Art, S. Knauer, R. Stauber, N. Sadri, A. Pont, R. J. Schneider, and H. Kleinert. 2009. Similar regulation of human inducible nitric-oxide synthase expression by different isoforms of the RNA-binding protein AUF1. *J Biol Chem* **284**: 2755-2766.
5. Abdelmohsen, K., K. Tominaga-Yamanaka, S. Srikantan, J. H. Yoon, M. J. Kang, and M. Gorospe. 2012. RNA-binding protein AUF1 represses Dicer expression. *Nucleic Acids Res* **40**: 11531-11544.
6. Banerjee, N., A. K. Bandyopadhyay, S. Dutta, J. K. Das, T. Roy Chowdhury, A. Bandyopadhyay, and A. K. Giri. 2017. Increased microRNA 21 expression contributes to

arsenic induced skin lesions, skin cancers and respiratory distress in chronically exposed individuals. *Toxicology* **378**: 10-16.
7. Varkonyi-Gasic, E., R. Wu, M. Wood, E. F. Walton, and R. P. Hellens. 2007. Protocol: a highly sensitive RT-PCR method for detection and quantification of microRNAs. *Plant Methods* **3**: 12.
8. Blake, M. S., K. H. Johnston, G. J. Russell-Jones, and E. C. Gotschlich. 1984. A rapid, sensitive method for detection of alkaline phosphatase-conjugated anti-antibody on Western blots. *Anal Biochem* **136**: 175-179.

Figure S1

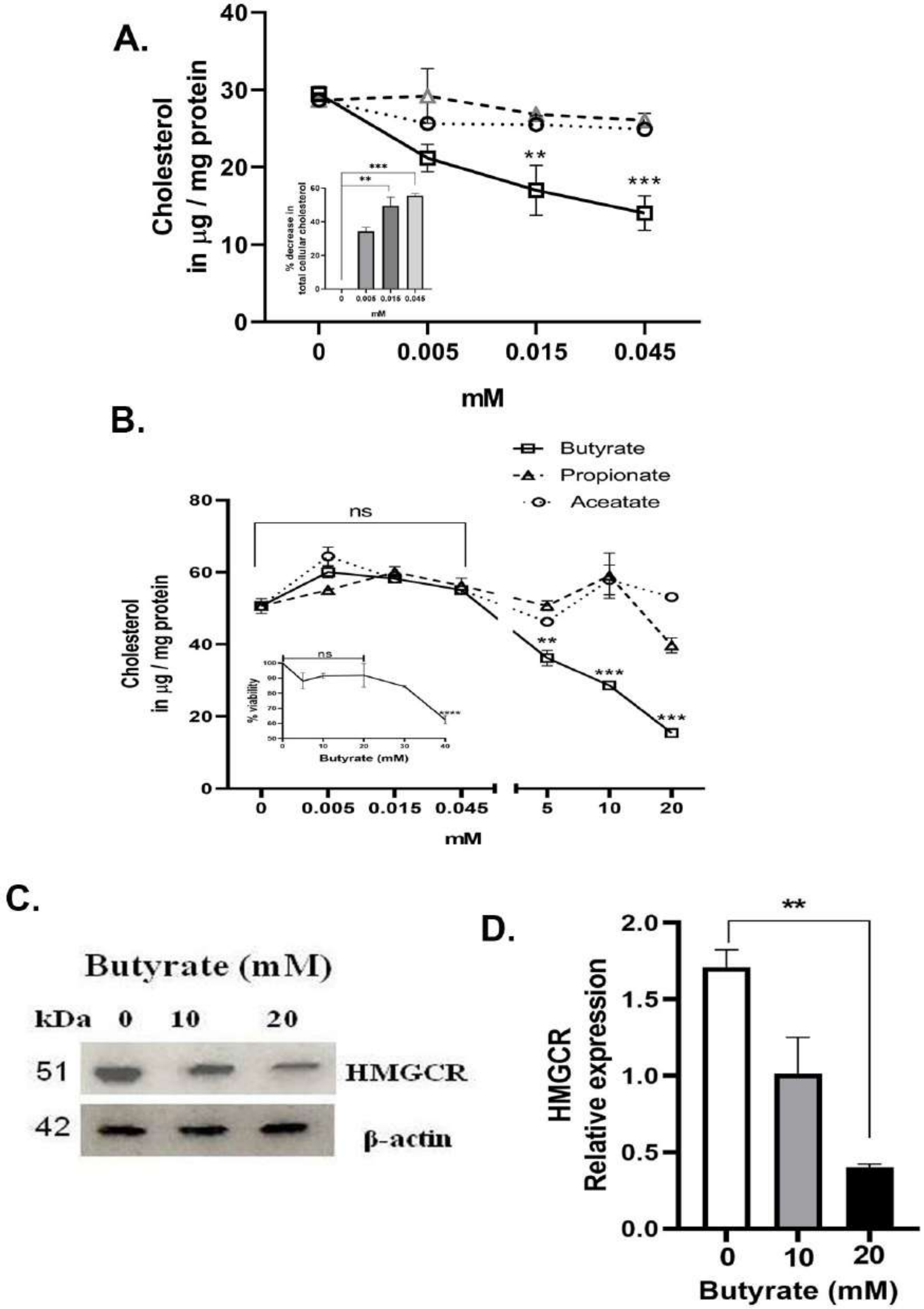

A.
Cholesterol
in µg / mg protein
40
30
20
10
0
**
***
0
0.005
0.015
0.045
mM
B.
Butyrate
Propionate
Aceatate
ns
80
60
40
20
0
**
***
***
Cholesterol
in µg / mg protein
% viability
Butyrate (mM)
0
0.005
0.015
0.045
5
10
20
mM
C.
Butyrate (mM)
kDa 0 10 20
51
HMGCR
42
β-actin
D.
**
HMGCR
Relative expression
2.0
1.5
1.0
0.5
0.0
0
10
20
Butyrate (mM)

Figure S2

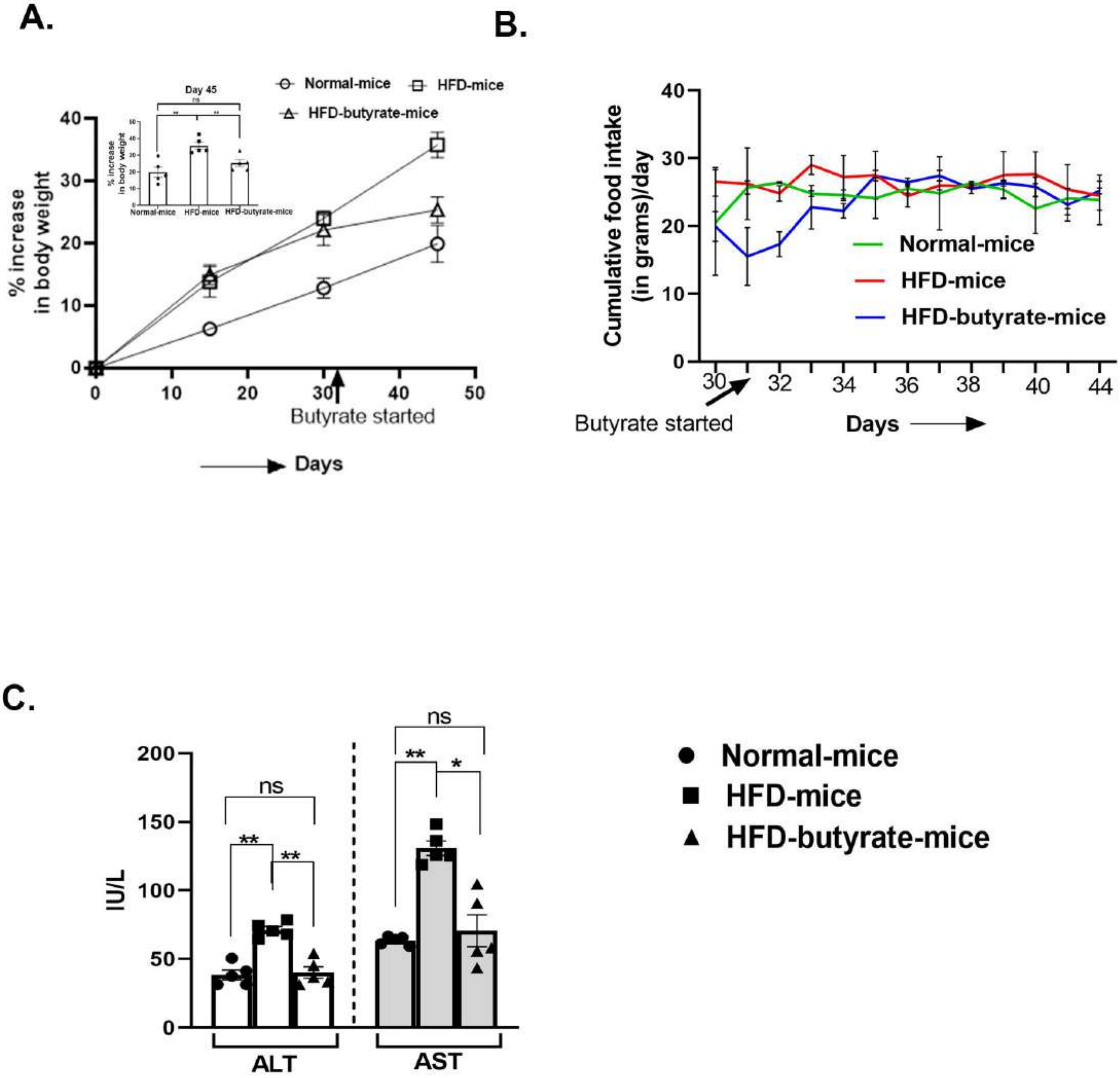
A.
Day 45
ns
% increase in body weight
Normal-mice
HFD-mice
HFD-butyrate-mice
% increase in body weight
Butyrate started
Days
B.
Cumulative food intake (in grams)/day
Normal-mice
HFD-mice
HFD-butyrate-mice
Butyrate started
Days
C.
IU/L
ns
**
*
ALT
AST
Normal-mice
HFD-mice
HFD-butyrate-mice

FIGURE S3

A

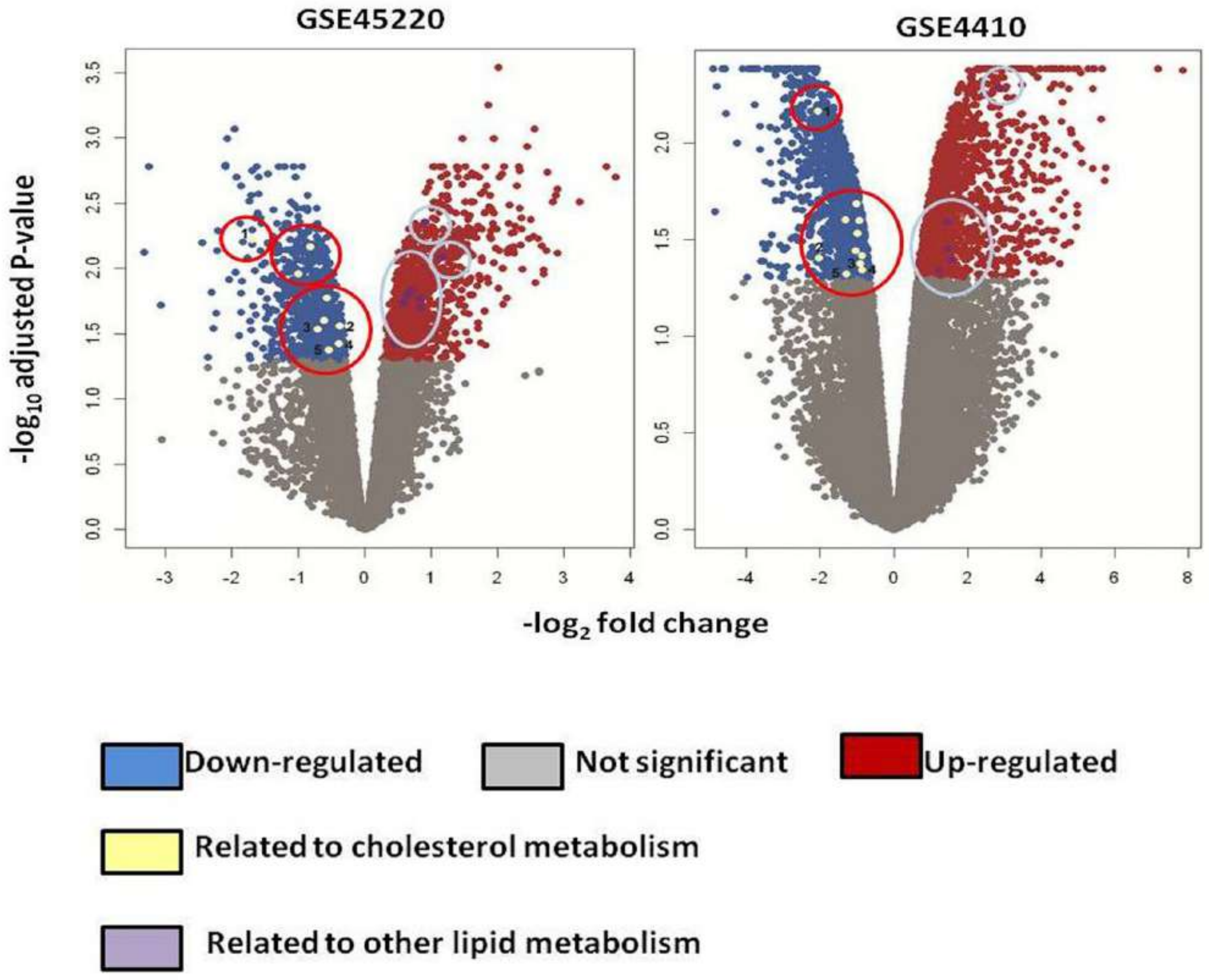

B

Common cholesterol metabolising genes down-regulated in both datasets

1. *Acat2* (Acetyl CoA acetyl transferase 2)
2. *Hmgcr* (HMG CoA reductase)
3. *Dhcr7* (7-dehydro cholesterol reductase)
4. *Hmgcs1* (HMG CoA synthase1)
5. *Dhcr24* (24-dehydro cholesterol reductase)

Figure S4

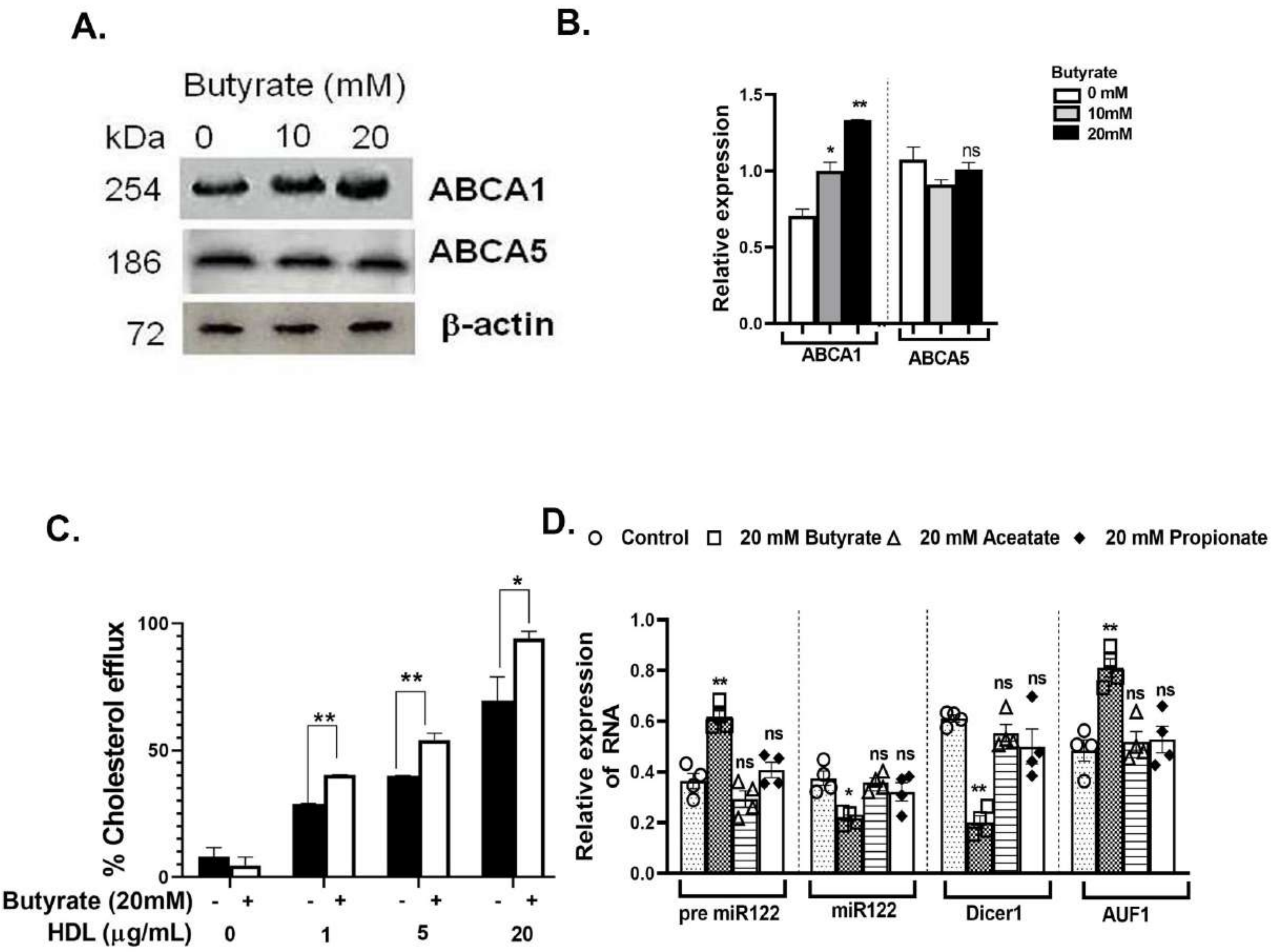
A.
Butyrate (mM)
kDa 0 10 20
254
ABCA1
186
ABCA5
72
β-actin
B.
Butyrate
0 mM
10mM
20mM
Relative expression
1.5
1.0
0.5
0.0
*
**
ns
ABCA1
ABCA5
C.
% Cholesterol efflux
100
50
0
**
**
*
Butyrate (20mM) - + - + - + - +
HDL (μg/mL) 0 1 5 20
D.
Control
20 mM Butyrate
20 mM Aceatate
20 mM Propionate
Relative expression of RNA
1.0
0.8
0.6
0.4
0.2
0.0
**
ns
*
ns
**
pre miR122
miR122
Dicer1
AUF1

Figure S5

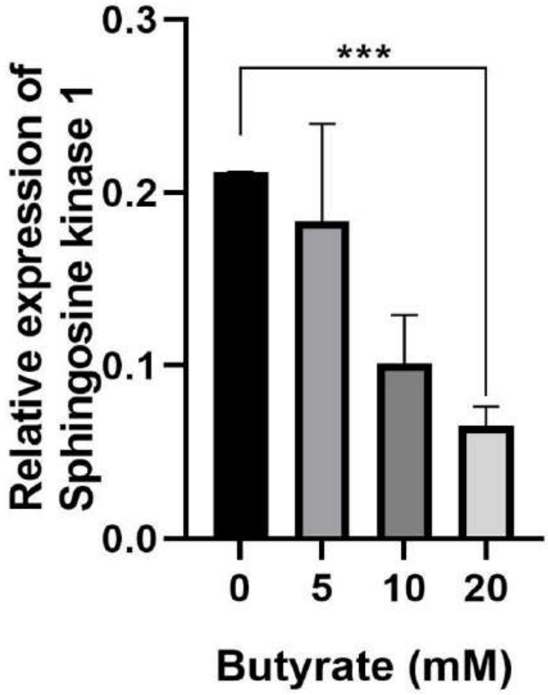

0.3
0.2
0.1
0.0
***
Relative expression of
Sphingosine kinase 1
0
5
10
20
Butyrate (mM)

Figure S6

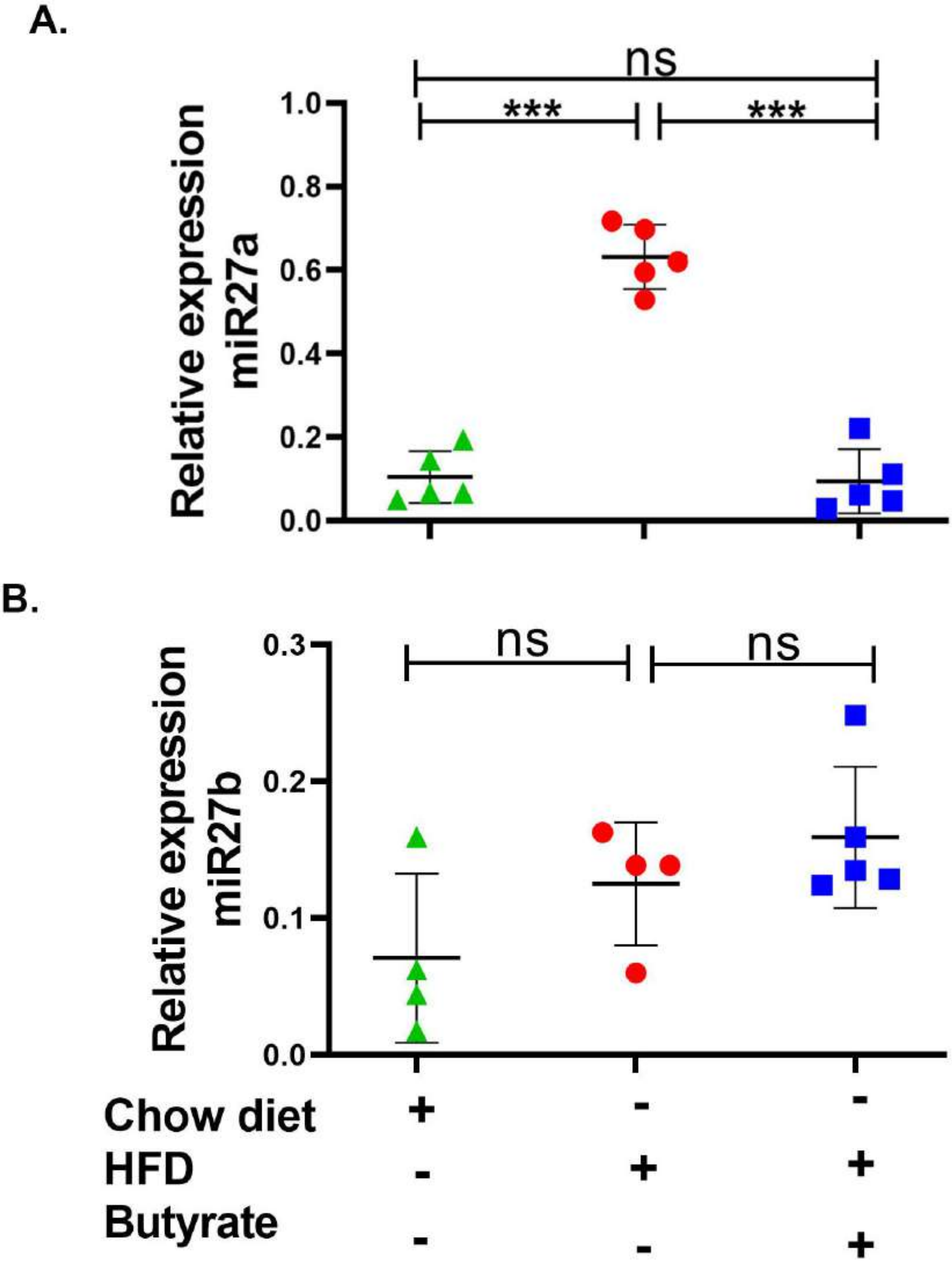
A.
ns
***
***
1.0
0.8
0.6
0.4
0.2
0.0
Relative expression
miR27a
B.
ns
ns
0.3
0.2
0.1
0.0
Relative expression
miR27b
Chow diet + - -
HFD - + +
Butyrate - - +

Figure S7

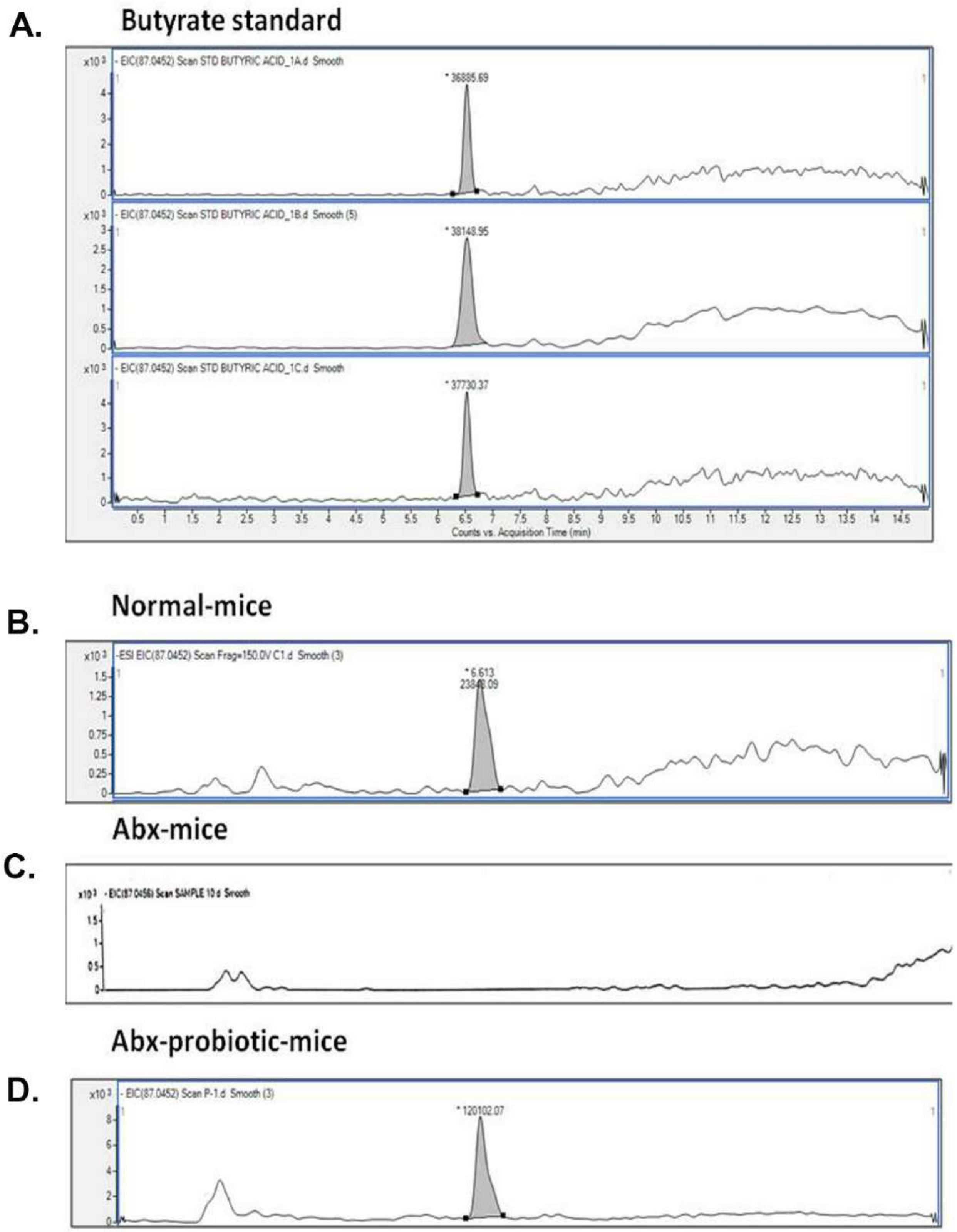
A.
Butyrate standard
x10 3
- EIC(87.0452) Scan STD BUTYRIC ACID_1A.d Smooth
* 36885.69
- EIC(87.0452) Scan STD BUTYRIC ACID_1B.d Smooth (5)
* 38148.95
- EIC(87.0452) Scan STD BUTYRIC ACID_1C.d Smooth
* 37730.37
Counts vs. Acquisition Time (min)
B.
Normal-mice
-ESI EIC(87.0452) Scan Frag=150.0V C1.d Smooth (3)
* 6.613
23848.09
Abx-mice
C.
Abx-probiotic-mice
D.
- EIC(87.0452) Scan P-1.d Smooth (3)
* 120102.07

Figure S8

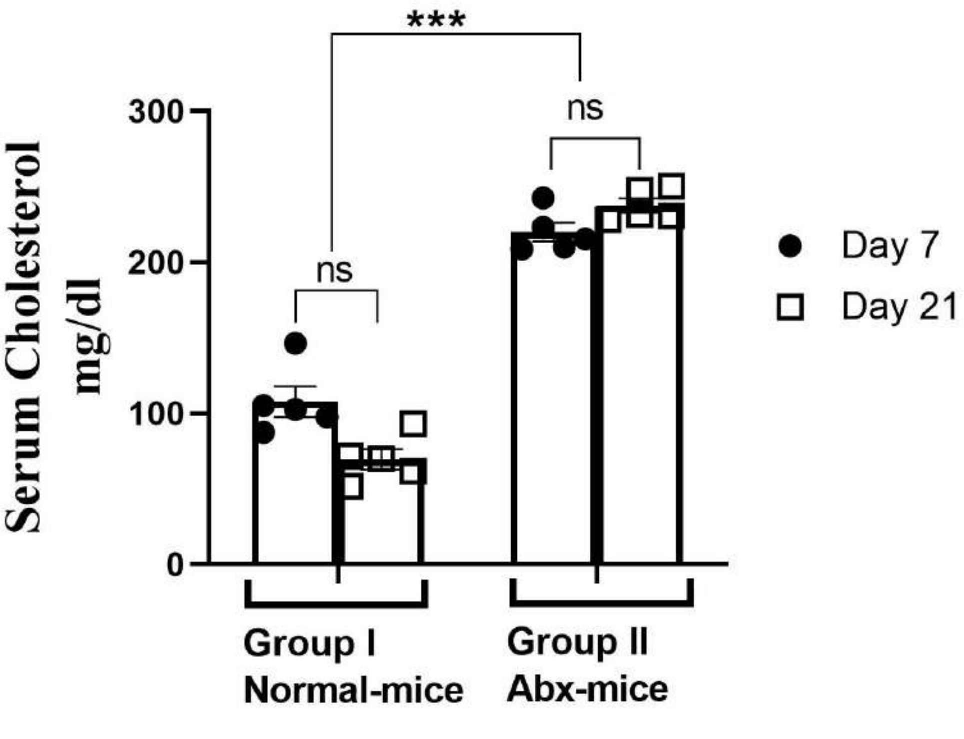

***
ns
ns
300
200
100
0
Serum Cholesterol
mg/dl
Day 7
Day 21
Group I
Normal-mice
Group II
Abx-mice

Figure S9

A.

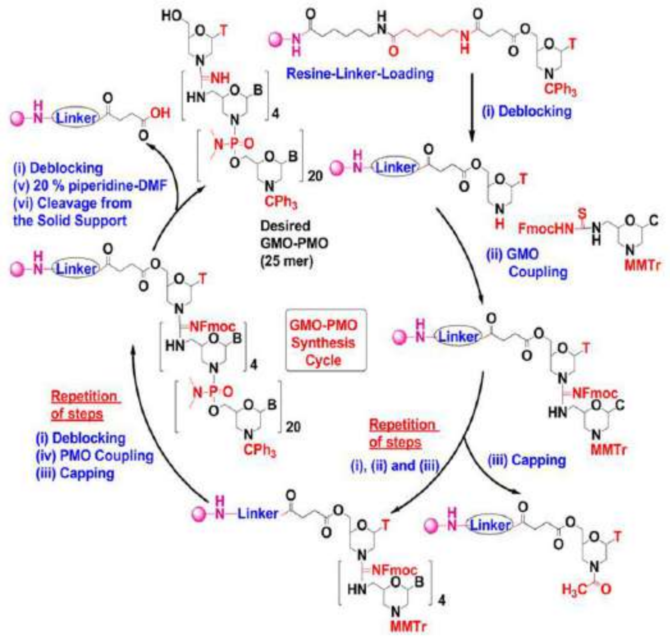

B.

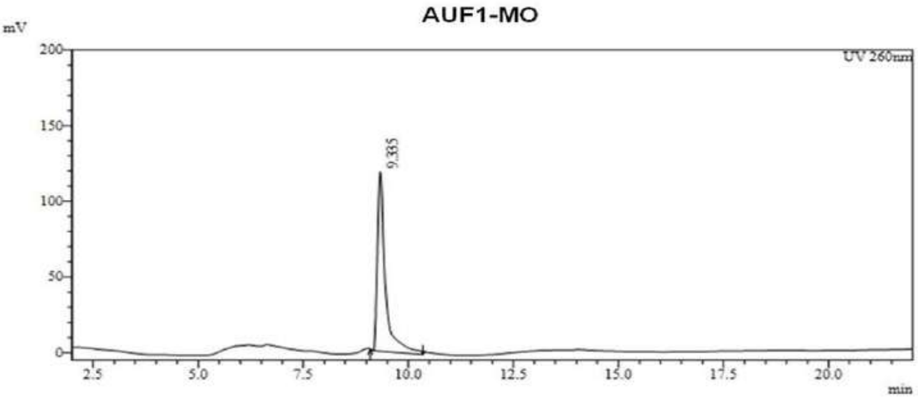

Peak Table

UV 260nm

Peak#	Ret. Time	Area	Height	Area%
1	9.335	1553739	118585	100.000
Total		1553739	118585	100.000

C.

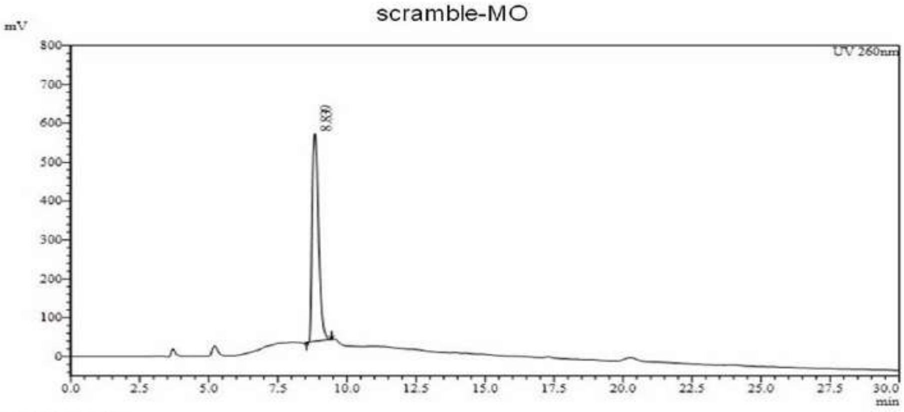

Peak Table

UV 260nm

Peak#	Ret. Time	Area	Height	Area%
1	8.839	8463967	533562	100.000
Total		8463967	533562	100.000

Figure S10

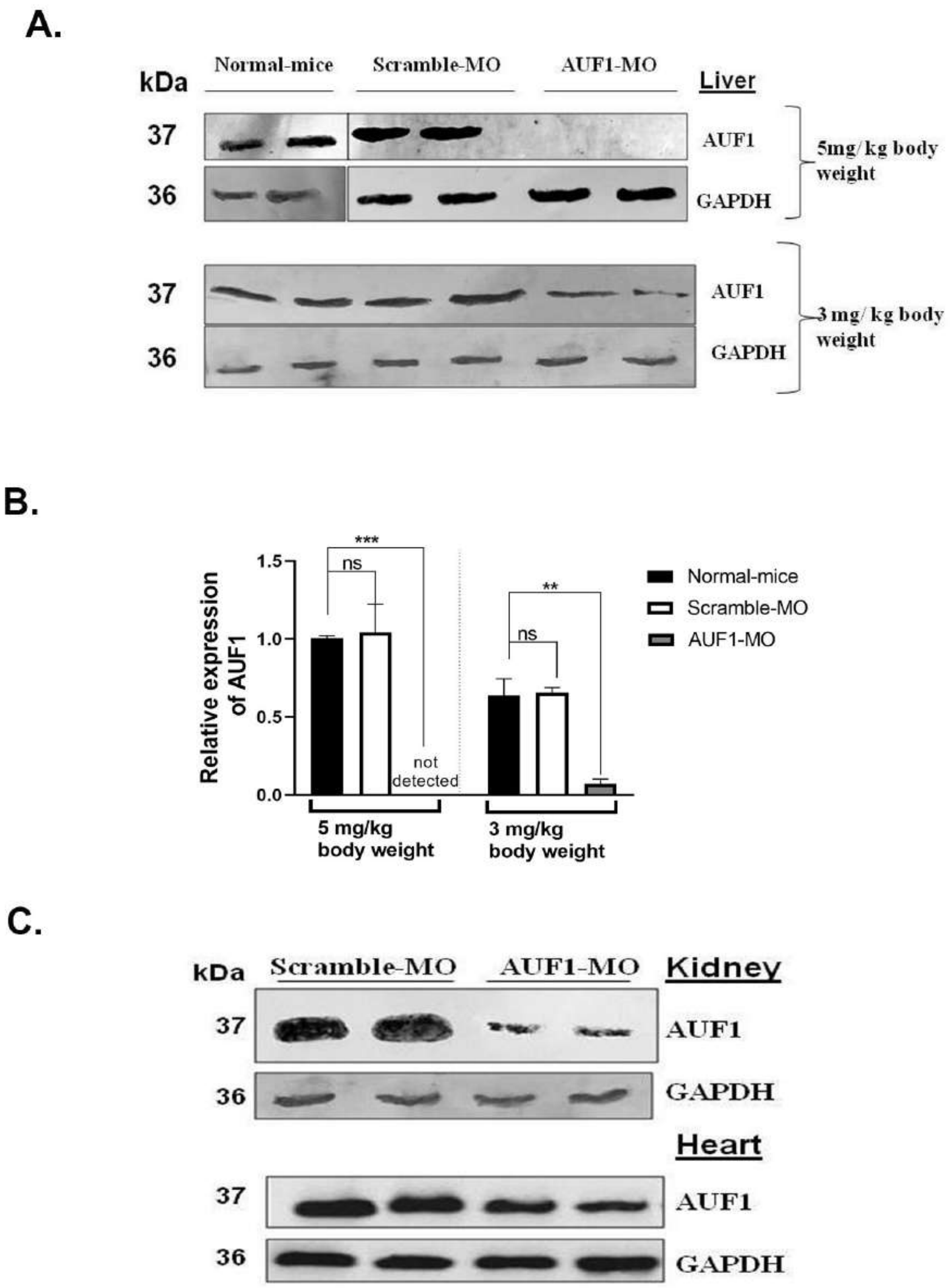
A.
kDa
Normal-mice
Scramble-MO
AUF1-MO
Liver
37
AUF1
36
GAPDH
5mg/ kg body weight
37
AUF1
36
GAPDH
3 mg/ kg body weight
B.
Relative expression of AUF1
1.5
1.0
0.5
0.0
***
ns
ns
**
not detected
5 mg/kg body weight
3 mg/kg body weight
Normal-mice
Scramble-MO
AUF1-MO
C.
kDa
Scramble-MO
AUF1-MO
Kidney
37
AUF1
36
GAPDH
Heart
37
AUF1
36
GAPDH
